# Exploring the relationship between patient-relevant outcomes and Alzheimer’s disease progression assessed using the clinical dementia rating scale: a systematic literature review

**DOI:** 10.3389/fneur.2023.1208802

**Published:** 2023-08-17

**Authors:** Jeffrey Cummings, Julie Hviid Hahn-Pedersen, Christian Stefan Eichinger, Caroline Freeman, Alice Clark, Luis Rafael Solís Tarazona, Krista Lanctôt

**Affiliations:** ^1^Chambers-Grundy Center for Transformative Neuroscience, Department of Brain Health, School of Integrated Health Sciences, University of Nevada, Las Vegas, NV, United States; ^2^Novo Nordisk A/S, Søborg, Denmark; ^3^Oxford PharmaGenesis, Oxford, United Kingdom; ^4^Hurvitz Brain Sciences Program, Sunnybrook Research Institute, Toronto, ON, Canada; ^5^Department of Psychiatry, University of Toronto, Toronto, ON, Canada

**Keywords:** activities of daily living, Alzheimer’s disease, clinical dementia rating (CDR), comorbidity, healthcare costs, healthcare resource use, neuropsychiatric inventory (NPI), nursing home placement

## Abstract

**Background:**

People with Alzheimer’s disease (AD) have difficulties in performing activities of daily living (ADLs) as the disease progresses, commonly experience neuropsychiatric symptoms (NPS), and often have comorbidities such as cardiovascular disease. These factors all contribute to a requirement for care and considerable healthcare costs in AD. The Clinical Dementia Rating (CDR) scale is a widely used measure of dementia staging, but the correlations between scores on this scale and patient-/care partner-relevant outcomes have not been characterized fully. We conducted a systematic literature review to address this evidence gap.

**Methods:**

Embase, MEDLINE, and the Cochrane Library were searched September 13, 2022, to identify published studies (no restriction by date or country) in populations with mild cognitive impairment due to AD or AD dementia. Studies of interest reported data on the relationships between CDR Global or CDR–Sum of Boxes (CDR-SB) scores and outcomes including NPS, comorbidities, ADLs, nursing home placement, healthcare costs, and resource use.

**Results:**

Overall, 58 studies met the inclusion criteria (42 focusing on comorbidities, 14 on ADLs or dependence, five on nursing home placement, and six on economic outcomes). CDR/CDR-SB scores were correlated with the frequency of multiple NPS and with total scores on the Neuropsychiatric Inventory. For cardiovascular comorbidities, no single risk factor was consistently linked to AD progression. Increasing CDR/CDR-SB scores were correlated with decline in multiple different measures of ADLs and were also associated with nursing home placement and increasing costs of care.

**Conclusion:**

NPS, ADLs, and costs of care are clearly linked to AD progression, as measured using CDR Global or CDR-SB scores, from the earliest stages of disease. This indicates that scores derived from the CDR are a meaningful way to describe the severity and burden of AD for patients and care partners across disease stages.

## 1. Introduction

In Alzheimer’s disease (AD), ongoing cognitive decline leads to difficulties in performing instrumental activities of daily living (IADLs), such as meal preparation, shopping, and household tasks, and ultimately to inability to perform basic activities of daily living (ADLs) such as dressing, bathing, and eating (1). AD is also associated with neuropsychiatric symptoms (NPS), which may manifest at early stages of the disease. Symptoms such as depression and apathy can herald the onset of the disease, with other NPS such as delusions and hallucinations typically appearing at advanced stages (2).

With increasing impairment in ADLs and mounting behavioral changes, people with AD require more care as the disease progresses. In many cases, a family member or spouse acts as a care partner for the individual affected by AD, but there may be a requirement for formal care, particularly in later disease stages (3). When care requirements cannot be met in the home, or when care partners are no longer able to act as the sole provider of care, individuals with AD may require admission to an assisted living residence, a nursing home, or other care facility (4).

AD is associated with considerable healthcare costs (5). The formal costs of AD are attributable in large part to the cost of nursing home placement; informal costs include care contributions made by care partners and employment opportunity losses (6, 7). AD is more prevalent with advancing age (8), and many patients have comorbidities, which require the use of additional healthcare resources (9, 10). Comorbidities have been implicated in the development and progression of AD, although the causality of these relationships remain to be fully elucidated (11).

The Clinical Dementia Rating (CDR) scale is commonly used to define AD stages and disease progression in research settings and clinical trials, but it is infrequently used in clinical practice. CDR Global and CDR-Sum of Boxes (CDR-SB) scores are derived from severity scores in six domains (see Measurement scales in the Materials and methods section), with higher scores indicating more severe AD (12). The specific impacts of AD can be quantified using various patient-, physician-, or care partner-reported outcome measures, including ADL and IADL questionnaires, and the Neuropsychiatric Inventory (NPI), which is used to assess the type, severity, and number of NPS (13). The relationships between these measures and the CDR scale, and their impact on costs as patients progress through AD stages, are incompletely characterized. A deeper understanding of these associations is vital to understand how CDR-SB and CDR Global scores relate to outcomes in clinical practice and costs of care, and the extrapolation of clinical trial data. Quantifying the impacts of reaching a particular AD stage may help to identify the potential benefits of interventions that slow progression and allow assessment of the economic impact of treatment-related changes.

We conducted a systematic literature review (SLR) to assess how comorbidities, NPS, ADL declines, nursing home placement, and economic costs are linked to AD severity and progression, as measured by CDR Global or CDR-SB scores.

## 2. Materials and methods

### 2.1. Systematic literature review

The SLR was designed to identify relevant evidence in populations with AD (both mild cognitive impairment [MCI] due to AD and AD dementia), on the relationship between CDR-SB or CDR Global scores and outcomes. All study types, including randomized controlled trials (RCTs), observational studies, and case studies and reports, were considered relevant. Following consultation with a panel of clinical experts, outcomes of specific interest were NPS, cardiovascular disease (CVD), epilepsy, bedsores, infections, incontinence, obstructive sleep apnea, falls, ADLs, dependence, nursing home placement, economic consequences, and healthcare resource use.

Both journal and congress publications were considered relevant. Primary publications and SLRs or meta-analyses were included, but narrative reviews were not. Only English language publications were included, but there was no restriction by date or geography.

The study protocol was designed and conducted in line with the 2020 Preferred Reporting Items for Systematic Reviews and Meta-Analyses (PRISMA) guidelines (14), and registered with PROSPERO (registration number: CRD42023392801). Searches of Embase, MEDLINE, and the Cochrane Library were conducted on September 13, 2022. Titles and abstracts were screened by one reviewer to determine whether they met the eligibility criteria (Table 1). All publications meeting the criteria were obtained as full articles and reassessed against the eligibility criteria.

**Table 1:** Eligibility criteria for the systematic literature review.

Populations	Patients with Alzheimer’s disease^a^ (from mild cognitive impairment to severe dementia of the Alzheimer’s type); this includes (1): MCI of the Alzheimer’s type (2); mild dementia of the Alzheimer’s type (3); moderate dementia of the Alzheimer’s type (4); severe dementia of the Alzheimer’s type
Interventions	Any or none
Comparators	Any or none
Outcomes	Outcomes reported alongside Clinical Dementia Rating scale Sum of Boxes (CDR-SB) score:^a^ Psychiatric diseases Depression Epilepsy Bedsores Cardiovascular disease Coronary disease Myocardial infarction Heart failure Cerebrovascular disease Stroke Peripheral vascular disease Infection Incontinence Obstructive sleep apnea Any other comorbidity reported alongside CDR-SB score, ^a^ or as a risk factor for progression measured by the CDR-SB^a^ Falls Nursing home placement Scores on the Dependence Scale Activities of daily living Economic outcomes, including data from economic modeling exercises Healthcare resource use
Study design	All study types, including randomized controlled trials, observational studies,^b^ and case studies and reports Exclusions: Animal/*in vitro* studies
Date restrictions	No restrictions
Language restrictions	English language
Publication type	All primary publications and systematic literature reviews/meta-analyses Exclusions: Reviews^c^/editorials
Country	Not restricted

a Studies reporting CDR scores were identified using search terms for CDR-SB and were also considered relevant.

b All study designs and data sources for observational studies were considered relevant.

c Narrative reviews and opinion articles.

CDR, Clinical Dementia Rating; CDR-SB, Clinical Dementia Rating-Sum of Boxes; MCI, mild cognitive impairment.

### 2.2. Data extraction

Detailed data, including study setting, methods, patient characteristics, and study results, were entered into a data extraction table, and quality checked by an independent reviewer.

Some studies reported longitudinal relationships between CDR-SB/CDR score progression and the progression of outcomes, whereas others reported cross-sectional data examining outcomes in different stages of AD severity. A third type of study reported how disease severity at baseline affected subsequent progression or how baseline characteristics affected AD progression.

### 2.3. Measurement scales

The CDR scale comprises six domains: memory, orientation, judgement and problem solving, community affairs, home and hobbies, and personal care (12), each of which is assigned a score of 0, 0.5, 1.0, 2.0, or 3.0.

CDR Global scores (referred to hereafter throughout this review as CDR scores for brevity) reflecting dementia severity, are calculated from the domain scores using an algorithm, which generates the following categories of severity:

- CDR 0 – no dementia
- CDR 0.5 – questionable dementia (can also be classified as MCI)
- CDR 1.0 – mild dementia
- CDR 2.0 – moderate dementia
- CDR 3.0 – severe dementia.

The CDR-SB score is derived by adding the scores of the six domains; the total score ranges between 0 and 18 (12).

The original 10-item version of the NPI (15) includes delusions, hallucinations, agitation/aggression, depression, anxiety, euphoria, apathy, disinhibition, irritability, and motor disturbances (13); night-time behavior disturbances, and appetite and eating abnormalities were later added (16). Each of the 12 items is scored on scales for frequency (1–4), severity (0–3), and distress (0–5). Individual scores for each domain are calculated as frequency × severity, and the total NPI score is the sum of the domain scores (13).

Various measures to assess ADLs and IADLs are used in AD research and clinical practice. Table 2 summarizes the scales included in this manuscript.

**Table 2:** Summary of relevant ADL measurement scales.

Scale	Details	Score range and interpretation
ADL scale (17)	Score of 1 (independence) or 0 (dependence) assigned on six functions: bathing, dressing, toileting, transferring, continence, and feeding (18)	Total score summed from function scores 6 – full function 4 – moderate impairment ≤ 2 – severe functional impairment (18)
IADL scale (19)	Score of 1 (greater independence) or 0 (lesser independence) assigned on eight functions: ability to use telephone, shopping, food preparation, housekeeping, laundry, mode of transportation, responsibility for own medications, and ability to handle finances (20)	Total score summed from function scores 8 – high function 0 – low function (20)
PSMS (19)	Scores between 1 (no impairment) and 5 (severe impairment) (21) assigned to six domains: toilet, feeding, dressing, grooming, physical ambulation, and bathing (22)	Maximum total score is 30 ≥ 6 – intact basic ADL (21)
ADCS-ADL (23)	Scores between 0 (total independence) and 4 (total dependence) assigned to 23 items (six basic ADLs and 17 IADLs) (24)	Total score summed from item scores Score range of 0–78, with a lower score indicating greater dependence (24)
A-IADL-Q (short version) (25)	Scores between 0 (no difficulty) and 4 (cannot perform the activity) assigned to 30 everyday activities	Total scores, which are calculated using item response theory, are in the approximate range of 20–80, with higher scores representing better daily functioning (26)
FAQ (27)	Scores between 0 (normal) and 3 (dependent) assigned to 10 daily activities, such as preparing a balanced meal and keeping track of current events (28)	Total score summed from item scores; range of 0–30 ≤ 9 (dependent in ≥3 activities) – impaired function and possible cognitive impairment (28)
FAST (29)	Assessment on AD severity scale from level 1 (normal) to level 7 (most severe level of functional impairment). Level 6 has five sub-scales, and level 7 has six sub-scales (30)	1 – normal aging 2 – possible MCI 3 – MCI 4 – mild dementia 5 – moderate dementia 6 – moderately severe dementia 7 – severe dementia (30)
Dependence scale (31)	Scores assigned to 13 items relating to ADLs and IADLs; 11 items scored as yes (1)/no (0) and two items scored as never (0)/occasionally (1)/frequently (2) (32)	Total score summed from item scores; range of 0–15, with higher scores indicating greater dependence (32)

AD, Alzheimer’s disease; ADCS-ADL, Alzheimer’s Disease Cooperative Study–Activities of Daily Living scale; ADL, activities of daily living; A-IADL-Q, Amsterdam Instrumental Activities of Daily Living Questionnaire; FAQ, Functional Activities Questionnaire; FAST, Functional Assessment Staging Tool; IADL, instrumental activities of daily living; MCI, mild cognitive impairment; PSMS, Physical Self-Maintenance Scale.

## 3. Results

### 3.1. Search results

**Figure 1:**
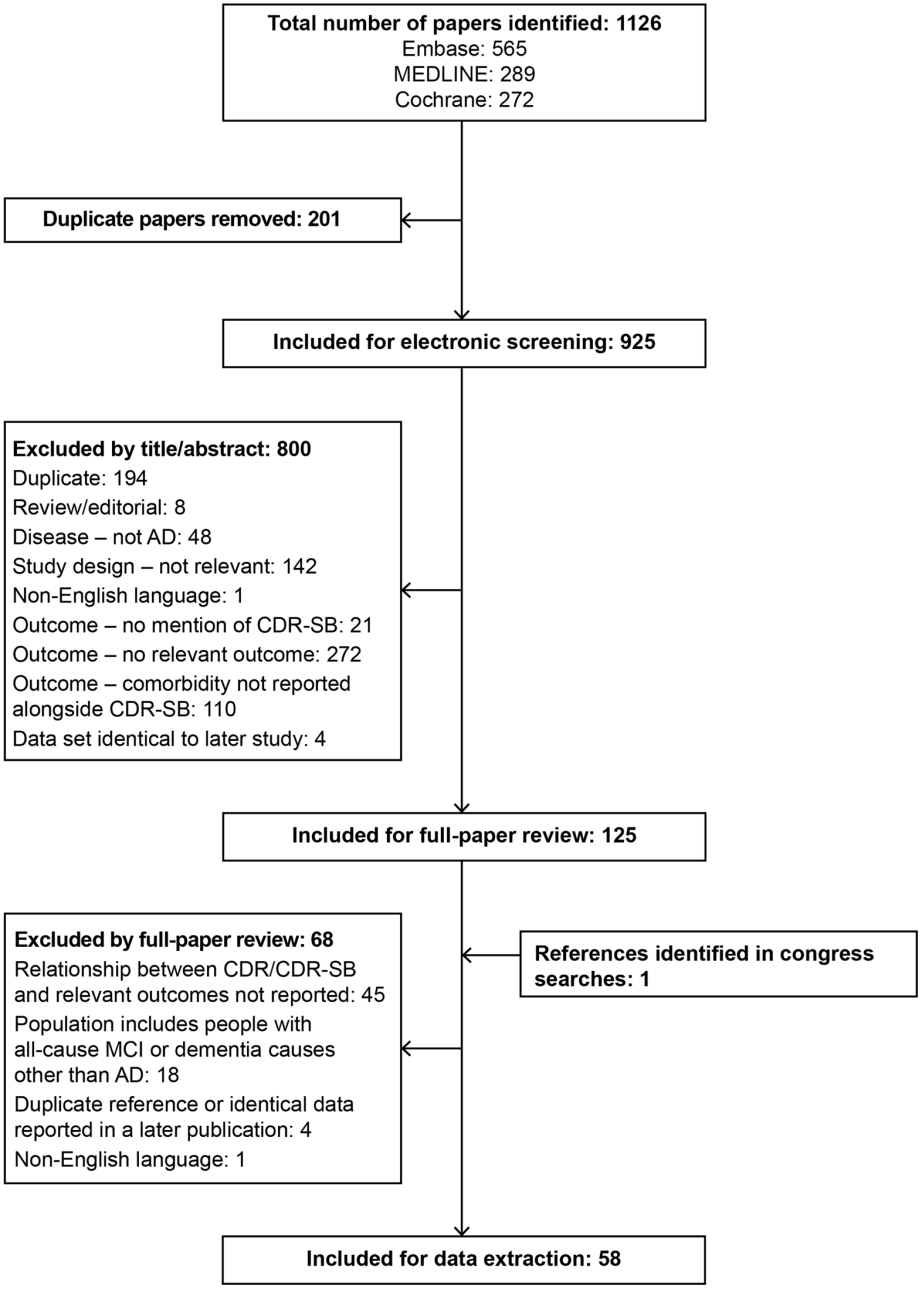
PRISMA diagram for the SLR. AD, Alzheimer’s disease; CDR, Clinical Dementia Rating; CDR-SB, Clinical Dementia Rating–Sum of Boxes; MCI, mild cognitive impairment; SLR, systematic literature review.

In total, 925 references were included for screening by abstract and title, resulting in 125 references included for full paper review (see Figure 1 for PRISMA flow diagram). In total, 57 references met the inclusion criteria at full paper review and were included for data extraction, along with one additional relevant reference that was identified in supplementary searches, resulting in 58 studies in total. Overall, 42 studies reported data on comorbidities (33–74), Twelve studies reported data on ADLs (21, 38, 49, 53, 62, 65, 67, 68, 75–78), two reported data on dependence (79, 80), five reported data on nursing home placement (68, 81–84), and six reported data on economic outcomes or resource use (68, 85–89). No studies reported data on bedsores, infections, obstructive sleep apnea, or falls.

### 3.2. Data sources and study characteristics

Nineteen studies reported data from Asia (China, India, Japan, Singapore, South Korea, Taiwan, and Thailand) (33, 38, 40–43, 57, 58, 60, 61, 63, 67, 73, 74, 76, 78, 82, 83, 87), 17 reported data from Europe (Finland, France, Germany, Italy, Norway, Spain, Turkey, and the UK) (34–37, 39, 44, 48–50, 53, 54, 56, 62, 80, 85, 88, 89), and 12 reported data from North America, all of which were from the USA (21, 51, 52, 55, 59, 64, 65, 68–72). Five studies reported data from multiple countries (75, 77, 79, 81, 86), three reported data from South America (Brazil) (45–47), and two publications did not report the country (66, 84). More than two-thirds of the studies used primary data (39 studies); the remainder were conducted using secondary data, with the exception of one study with an unclear design (80). Individuals with AD were typically recruited from memory clinics, neurology departments, or hospitals, or as part of cohort studies of ageing, dementia, or AD. A small number of studies analyzed data collected in RCTs.

### 3.3. AD diagnosis and staging

Most studies diagnosed AD according to standard clinical criteria, typically the National Institute of Neurological and Communicative Disorders and Stroke−Alzheimer’s Disease and Related Disorders Association (NINCDS-ADRDA) criteria (90). However, only five studies (34, 49, 72, 75, 77) confirmed the diagnosis of AD using biomarkers. Four studies measured β-amyloid and/or tau in cerebrospinal fluid (34, 49, 75, 77), one of which detected amyloid deposits via positron emission tomography scans as an alternative method (75). The fifth study detected amyloid deposits using immunohistochemistry *post mortem* (72).

### 3.4. Comorbidities

#### 3.4.1. Neuropsychiatric symptoms

Increasing AD severity, as measured using CDR or CDR-SB scores, was predictably linked to greater occurrence of NPS, but there was variation in which NPS were most closely associated with progression to more advanced stages of AD. Differences may be attributable in part to sample sizes, residential setting, comorbidities and concomitant medications, and data collection strategies.

Overall 22 studies reported data on NPS (33, 34, 36–39, 41, 42, 44, 53, 54, 56, 57, 62, 65–67, 69, 71–74), of which the majority used the NPI.

**Figure 2:**
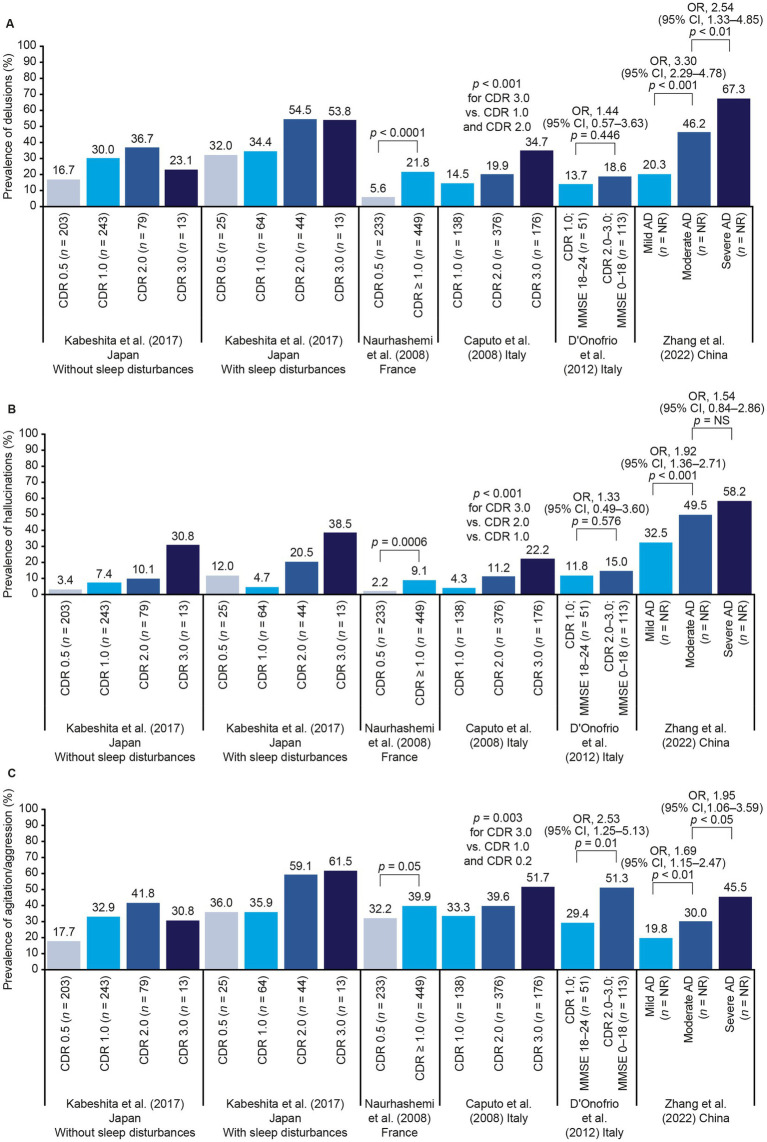

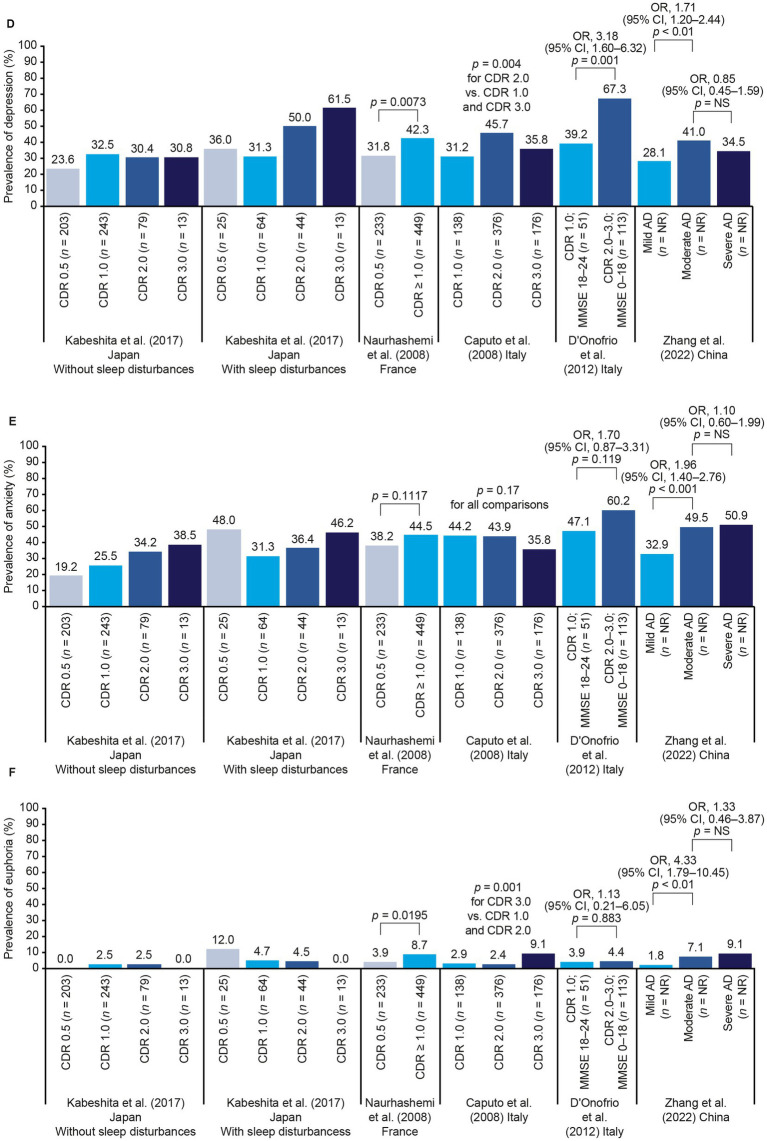

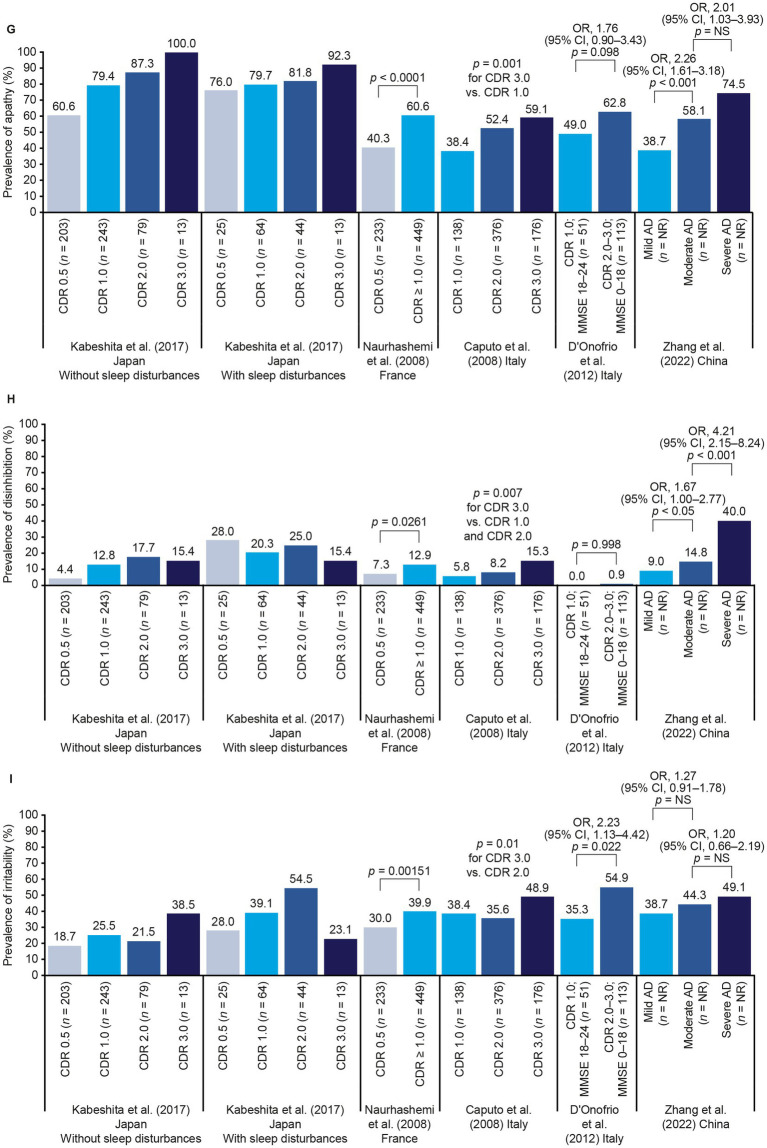

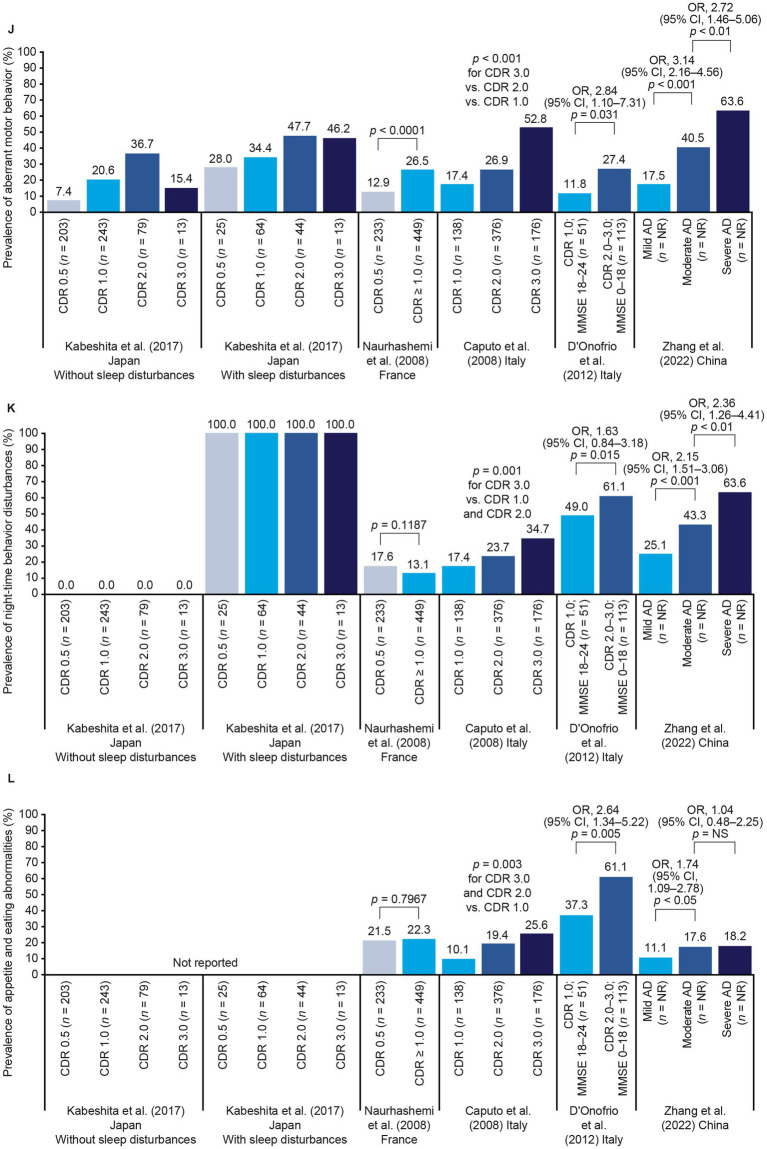
NPS frequency by AD stage. Panels **(A–L)** show data for each individual NPS (see labels on y-axes). Where *p* values are not shown, statistical tests were not conducted. *p* values across multiple categories represent tests of linear trends. Zhang et al. (74) used the CDR to determine disease severity, but did not specify exactly how each AD stage was defined (74). AD, Alzheimer’s disease; CDR, Clinical Dementia Rating; CI, confidence interval; MMSE, Mini Mental State Examination; NPS, neuropsychiatric symptom(s); NR, not reported; OR, odds ratio.

Figure 2 shows data from the studies that reported the prevalence of specific NPS by AD stage (37, 44, 57, 62, 74). The frequency of these symptoms and their link to disease severity differed across studies. For delusions, hallucinations, agitation/aggression, apathy, disinhibition, aberrant motor behavior, night-time behavior disturbances, and appetite and eating abnormalities, there was a stepwise relationship between prevalence and AD severity in most studies. For depression, anxiety, euphoria, and irritability, there were smaller differences in prevalence across AD stages.

Another subset of studies reported links between NPS and disease progression (Supplementary Table 2). Barca et al. found that CDR-SB score and trajectories of depressive symptoms were correlated over time (34). Caroline et al. found that individuals with mild-to-moderate AD dementia who experienced fast CDR-SB score progression had a higher prevalence of delusions, depression, anxiety, apathy, and aberrant motor behavior at baseline than those with slow progression (38). Hallikainen et al. found that delusions and euphoria predicted AD progression, as measured using CDR-SB scores, in multivariate analyses, and AD severity during follow-up was associated with the longitudinal occurrence of hallucinations, delusions, agitation, apathy, aberrant motor behavior, and sleep and appetite disturbances (54). Some inconsistencies were noted among the studies identified. Wadsworth et al. found that CDR-SB scores were associated only with apathy at baseline and were not associated with any NPS over follow-up (71), and Breitve et al. found no link between CDR or CDR-SB scores and patient-or care partner-reported anxiety (36).

Charernboon et al. found that people with higher CDR scores tended to have significantly more NPS than those with lower scores (41), and Bandyopadhyay et al. found a weak positive correlation between CDR score and number of NPS (Supplementary Table 2) (33).

Both Hallikainen et al. (53) and Naurhashemi et al. (62) found that people with higher CDR scores tended to have higher NPI scores, but Jenner et al. (56) found no correlation between CDR scores and NPI severity scores. Tschanz et al. reported only a weak correlation between CDR-SB and NPI scores over a mean of 3.8 years’ follow-up (69), Caroline et al. found that baseline NPI score was a significant predictor of fast CDR-SB score progression over 1 year (38). In contrast, Tay et al. found no significant differences in baseline NPI score between people who did and did not progress in terms of CDR-SB score over 1 year; those who progressed experienced significantly greater changes in their NPI score over this time (67).

The CDR/CDR-SB assesses cognition and function but does not include any measures or questions regarding behavior. Collecting behavioral and NPS data with alternative tools such as the NPI assists in a more robust characterization of the patient’s clinical status.

#### 3.4.2. CVD

In total, 17 studies reported data on CVD or cardiovascular risk factors (35, 42, 43, 45–47, 49, 50, 55, 58–60, 64, 67, 68, 72, 73) (Supplementary Table 3). These studies present some evidence that the presence of cardiovascular risk factors can influence AD progression, but there was little concordance across studies in terms of which particular risk factors are associated with more severe AD stages or with faster disease progression.

**Figure 3:**
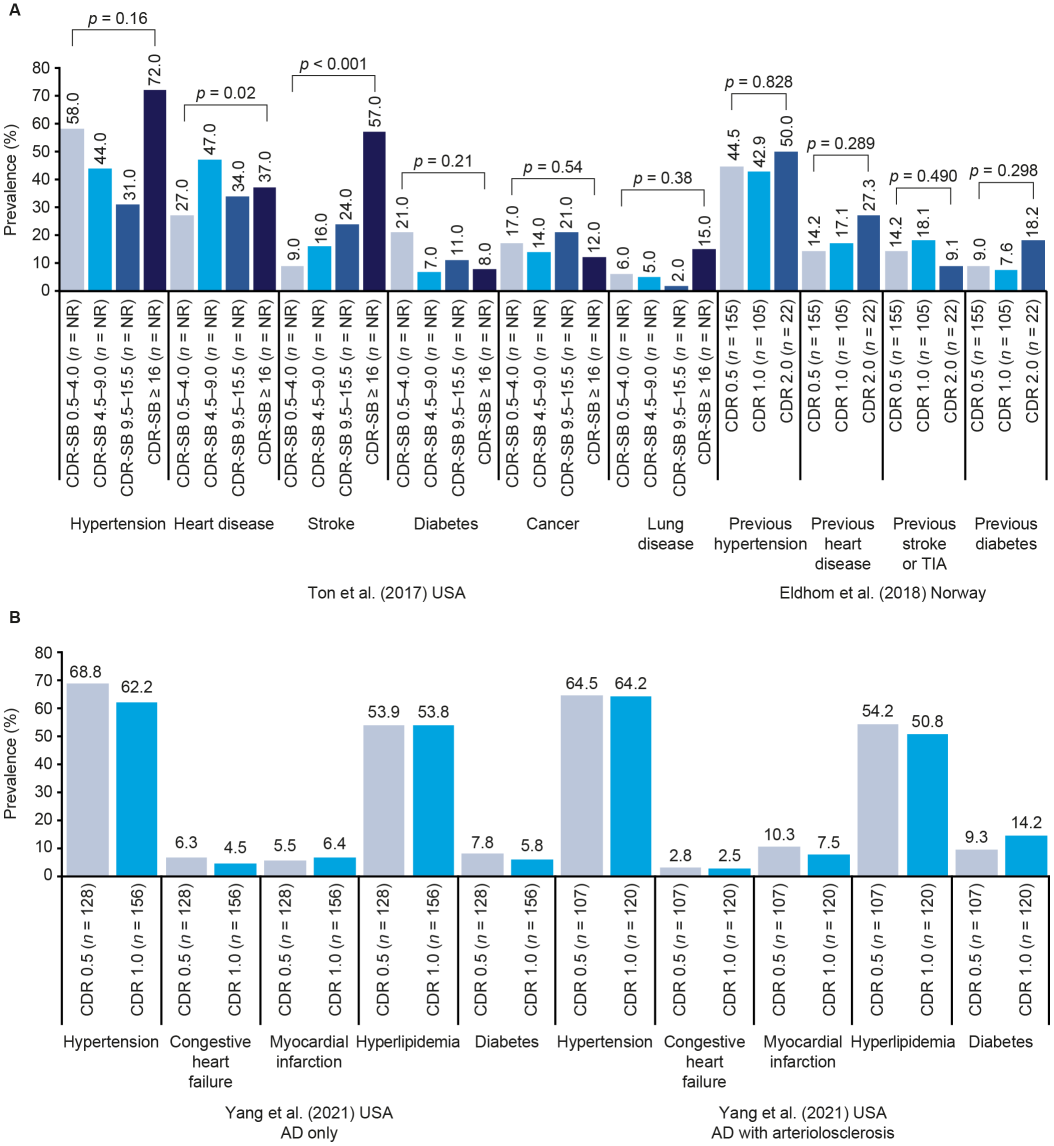
Prevalence of comorbidities by AD stage in Ton et al. (68) and Eldholm et al. (50) **(A)** and Yang et al. (72) **(B)**. *p* values across multiple categories represent tests of linear trends. Tests of statistical significance in Ton et al. (68) also include a group with normal cognition (data not shown). AD, Alzheimer’s disease; CDR, Clinical Dementia Rating; CDR-SB, Clinical Dementia Rating–Sum of Boxes; TIA, transient ischemic attack.

Figure 3 shows data from studies that reported the prevalence of various comorbidities by AD stage, as denoted by categories of CDR or CDR-SB scores (50, 68, 72). The only clear difference between stages was in the study conducted by Ton et al. in which people with more severe AD dementia were relatively more likely to have experienced stroke; other conditions did not appear to be linked to AD stage (68). Of the other studies that reported data on multiple comorbidities, Lee et al. found no link between individual vascular risk factors and CDR-SB progression, but found that having three or more vascular risk factors (coronary heart disease, cardiac arrhythmia, cerebrovascular accident, hypertension, diabetes mellitus, obesity, smoking, or physical inactivity) was linked to progression (58). Mielke et al. found that baseline atrial fibrillation, high systolic blood pressure (as a categorical variable), angina, coronary artery bypass graft, diabetes, or receipt of antihypertensive medication, but not myocardial infarction or stroke, were linked to CDR-SB score progression (59).

#### 3.4.3. Other comorbidities of interest

Three publications reported cross-sectional data on AD severity and urinary incontinence. Na et al. conducted analyses adjusted for age and various AD severity and symptom scores, and found that urinary incontinence was significantly associated with CDR-SB score (odds ratio [OR], 1.56; 95% CI, 1.21–2.01; *p* < 0.05) among 464 people with AD dementia in South Korea (61); similar results were reported in a previous abstract by the same authors (63). Chang et al. found that scores on the Overactive Bladder Symptom Score questionnaire were not significantly associated with CDR or CDR-SB score in 43 people from Taiwan with AD dementia, but the individual symptom score of urge incontinence was highly correlated with CDR-SB score (*r* = 0.314; *p* < 0.05) (40).

Only one study that assessed epilepsy was identified; Voglein et al. assessed US National Alzheimer’s Coordinating Center data from 20,745 individuals, and found that those who experienced seizures had a higher mean CDR-SB score than those who did not (9.3 vs. 6.8; *p* < 0.0001) in a model adjusted for age and disease duration (70).

### 3.5. ADLs and dependence

Twelve studies assessed ADLs (Table 3), and these data demonstrated, not surprisingly, that CDR and CDR-SB scores were reliably correlated with decline in ADLs and IADLs, irrespective of the ADL measurement instrument used in the study.

**Table 3:** Studies reporting ADL data.

Study and country	Population	AD severity/staging measure	ADL measure	Results
Studies with longitudinal data				
Hotta et al. (76) Japan	N = 671 with AD dementia (stage NR)	CDR	PSMS IADL	“Tendency to decrease gently was seen in ADLs according to decline of CDR score in AD”^a^
Hallikainen et al. (53) Finland	*N* = 115 with AD (CDR 0.5 or CDR 1.0)	CDR and CDR-SB	ADCS-ADL	**Longitudinal progression of CDR-SB score and ADCS-ADL score** Correlation between CDR-SB score and ADCS-ADL score after 3 years: −0.817 (*p* < 0.001) **ADCS-ADL progression by baseline CDR score group** ADCS-ADL score, mean (SEM) Baseline CDR 0.5: 70.3 (0.6) CDR 1.0: 62.0 (1.1) *p* < 0.001 1 year CDR 0.5: 66.1 (0.9) CDR 1.0: 55.3 (1.6) *p* < 0.001 2 years CDR 0.5: 60.2 (1.4) CDR 1.0: 50.0 (1.9) *p* < 0.001 3 years CDR 0.5: 54.0 (1.9) CDR 1.0: 40.6 (2.3) *p* < 0.001
McDougall et al. (77) Multinational	*N* = 797 with AD (CDR 0.5 and MMSE ≥24) *n* = 104 with follow-up data	CDR-SB	FAQ	Correlation coefficient between CDR-SB and FAQ Baseline: 0.6 Change from baseline to week 104: 0.6
Tay et al. (67) Singapore	*N* = 96 (14 with MCI; 74 with AD dementia) *n* = 88 with follow-up	CDR-SB	IADL	IADL, mean (SD) Baseline Progressors^b^: 12.5 (3.4) Non-progressors: 15.6 (5.4) *p* = 0.008 1 year Progressors^b^: 9.1 (4.3) Non-progressors: 14.5 (5.5) *p* = 0.001 Change over 1 year’s follow-up Progressors^b^: −3.4 (4.3) Non-progressors: −8.2 (1.1) *p* = 0.059
Eldholm et al. (49) Norway	*N* = 282 with CDR 0.5, 1.0, or 2.0 (very mild, mild, or moderate AD dementia)	CDR and CDR-SB	IADL	**Longitudinal progression of CDR-SB score and IADL score** Correlation between change in IADL score and change in CDR-SB: −0.614 **Baseline IADL score, mean (SD), by subsequent CDR-SB progression** Intermediate or rapid progressors^c^ (*n* = 145): 0.76 (0.22) Slow progressors^d^ (*n* = 127): 0.82 (0.21) *p* = 0.030 **IADL scores and score changes by CDR group at baseline** IADL score, mean (SD) CDR 0.5: 0.90 (0.15) CDR 1.0: 0.69 (0.20) CDR 2.0: 0.45 (0.22) *p* < 0.001 Change in IADL score over follow-up (mean: 24 months), mean (SD) CDR 0.5: −0.12 (0.14) CDR 1.0: −0.14 (0.14) CDR 2.0: −0.13 (0.15) *p* = 0.472
Park et al. (21) USA	*N* = 267 with mild AD dementia (MMSE ≥20)	CDR-SB	PSMS IADL	**CDR-SB scores, mean (SD), according to ADLs at baseline** Unimpaired ADLs (*n* = 40) Baseline: 3.0 (1.2) 1 year follow-up: 4.2 (1.4) Annualized difference: 0.9 (0.95) Impaired ADLs^e^ (*n* = 227) Baseline: 5.1 (2.3) 1 year follow-up: 7.2 (3.6) Annualized difference: 1.7 (2.5) *p* = 0.11
Naurhashemi et al. (62) France	*N* = 682 with AD (CDR 0.5–3.0)	CDR	ADL IADL	**Rate of progression over follow-up, mean (SD), by CDR score at baseline** ADL score CDR 0.5: −0.51 (0.98) CDR ≥ 1.0: −1.34 (1.47) *p* < 0.0001 IADL score CDR 0.5: −1.30 (1.27) CDR ≥ 1.0: −1.18 (1.11) *p* = 0.4848 **Baseline data** ADL score, mean (SD) CDR 0.5: 5.85 (0.4) CDR ≥ 1.0: 5.22 (1.01) *p* < 0.0001 IADL score, mean (SD) CDR 0.5: 6.04 (1.67) CDR ≥ 1.0: 3.40 (1.91) *p* < 0.0001
Caroline et al. (38) China	*N* = 101 with mild/moderate AD dementia *n* = 94 with follow-up data	CDR-SB	IADL	In univariate analyses, IADL score at baseline was a significant predictor (*p* < 0.05) of fast progression^f^ over 1 year’s follow-up
Studies with cross-sectional data only				
Ton et al. (68) USA	*n* = 121 with amnestic MCI *n* = 174 with AD dementia	CDR-SB	ADL IADL	Difficulties with any ADL, % Amnestic MCI (CDR-SB 0.5–4.0): 29 Mild AD dementia (CDR-SB 4.5–9.0^g^): 36 Moderate AD dementia (CDR-SB 9.5–15.5): 73 Severe AD dementia (CDR-SB ≥ 16.0): 94 *p* < 0.01 (also includes group with normal cognition) Difficulties with any IADL, % Amnestic MCI (CDR-SB 0.5–4.0): 11 Mild AD dementia (CDR-SB 4.5–9.0): 57 Moderate AD dementia (CDR-SB 9.5–15.5): 78 Severe AD dementia (CDR-SB ≥ 16.0): 100 *p* < 0.01 (also includes group with normal cognition)
Royall et al. (65) USA	*N* = 70 with MCI or AD dementia (CDR 0.5, *n* = 47; CDR 1.0, *n* = 23)	CDR-SB	IADL	The coefficient d, a latent construct of IADL, was a predictor of CDR-SB score (*r* = −0.91; *p* ≤ 0.001)
Dubbelman et al. (75) Multinational	*N* = 799 with AD dementia	CDR	A-IADL-Q	A-IADL-Q scores were moderately correlated with CDR-SB^h^ scores (*r* = −0.55; 95% CI, −0.60 to −0.49)
Na et al. (78) South Korea	*N* = 464 with mild, moderate, or severe AD dementia	CDR and CDR-SB	FAST	Pearson correlation coefficients FAST and CDR Total sample: 0.83 Mild-to-moderate: 0.76 Severe: 0.70 *p* < 0.001 FAST and CDR-SB Total sample: 0.86 Mild-to-moderate: 0.83 Severe: 0.74 *p* < 0.001

AD, Alzheimer’s disease; ADCS-ADL, Alzheimer’s Disease Cooperative Study–Activities of Daily Living scale; ADL, activities of daily living; A-IADL-Q, Amsterdam Instrumental Activities of Daily Living Questionnaire; CDR, Clinical Dementia Rating; CDR-SB, Clinical Dementia Rating–Sum of Boxes; FAQ, Functional Activities Questionnaire; FAST, Functional Assessment Staging Tool; IADL, instrumental activities of daily living; MCI, mild cognitive impairment; MMSE, Mini Mental State Examination; PSMS, Physical Self-Maintenance Scale; SD, standard deviation; SEM, standard error of the mean.

a This is the exact wording used in the congress abstract; no supporting data are provided.

b Disease progression was defined as an increase ≥ 2 points from baseline on CDR-SB score.

c Intermediate or rapid progression was defined as > 1 change in CDR-SB score per year over follow-up.

d Slow progression was defined as < 1 change in CDR-SB score per year over follow-up.

e Impaired ADLs was defined as a total score on the PSMS > 6 and a total score on the IADL Scale > 9.

f Fast progression was defined as a decline of CDR-SB score of ≥ 2 points.

g Mild dementia is defined instead as CDR-SB 3.0–9.0 in figure legends in the publication; we have assumed that this is an error, because this overlaps with the CDR-SB score range for amnestic MCI (0.5–4.0).

h The score is separately referred to as CDR-SB and CDR in different places in the study publication (congress abstract).

Five studies assessed AD progression and ADLs longitudinally. Hotta et al. found that the ability to perform ADLs, assessed using the Physical Self-Maintenance Scale (PSMS) and the IADL Scale, decreased with increasing CDR score over time (76). Eldholm et al. (49), Hallikainen et al. (53), and McDougall et al. (77) found correlations between changes in CDR-SB scores and decline in ADLs as measured using the IADL Scale, the Alzheimer’s Disease Cooperative Study (ADCS)-ADL scale, and the Functional Activities Questionnaire (FAQ), respectively. Tay et al. found that IADL scores were significantly worse at both baseline and follow-up for people who progressed by 2 CDR-SB points or more from baseline over 1 year than for those who did not progress, although no significant differences were detected in terms of the extent of IADL score change (67).

Four studies used cross-sectional data. Royall et al. (65), Dubbelman et al. (75), and Ton et al. (68) found that IADL scores were correlated with CDR or CDR-SB scores; the latter study also found that ADL scores were correlated with CDR-SB scores. Na et al. found that both CDR and CDR-SB scores were correlated with Functional Assessment Staging Tool (FAST) stage (78).

Five studies assessed correlations at baseline and subsequent progression. Hallikainen et al. found that patients with a CDR score of 0.5 at baseline maintained better ADCS-ADL scores each year over 3 years of follow-up than those with a baseline CDR score of 1.0 (53). Naurhashemi et al. found that individuals with AD who had a CDR score of 1.0 or greater at baseline experienced significantly greater ADL decrements over the following 2 years than those with a baseline score of CDR 0.5; however, there was no significant difference between the groups in terms of changes in the ability to perform IADLs (62). Similarly, Eldholm et al. found no significant differences between annual IADL score changes in patients with baseline CDR scores of 0.5, 1.0, or 2.0, which correspond to MCI due to AD, mild AD dementia, and moderate AD dementia, respectively (49). Caroline et al. found that baseline IADLs were a predictor of fast CDR-SB score progression (38), but Park et al. classified patients as having impaired or unimpaired ADLs at baseline using the PSMS and IADL Scale, and found no difference between groups in terms of CDR-SB score decline over the following year (21).

Two studies reported a correlation between scores on the Dependence Scale and CDR-SB scores, using cross-sectional data. Cohen et al. found that a 1-point change in CDR-SB score corresponded to a change of 0.68 points on the Dependence Scale in a multinational cohort with MCI or mild-to-moderate AD dementia (79), whereas Jones et al. found a Dependence Scale score increase of 0.47 for a 1-point increase in CDR-SB score in a UK population with AD dementia (80). For context, total score ranges are broadly similar for the CDR-SB and the Dependence Scale (0–18 and 0–15, respectively; Table 2).

### 3.6. Nursing home placement

Five studies examined associations between the risk of nursing home placement and CDR-SB or CDR scores (68, 81–84). AD severity, denoted using CDR or CDR-SB scores, was linked to nursing home placement in studies reporting cross-sectional and short-term longitudinal data.

Park et al. assessed the risk of nursing home placement over approximately 3 years’ follow-up in people recently diagnosed with AD in South Korea (*n* = 2,470 with baseline data; *n* = 816 with follow-up data) (83). According to the results of Cox proportional hazards models to identify predictors of nursing home placement, baseline CDR-SB score was not a significant predictor (hazard ratio [HR], 1.04; 95% CI, 0.99–1.09; *p =* 0.05), but annual CDR-SB score change was a predictor (HR, 1.15; 95% CI, 1.06–1.23; *p* < 0.01) (21). Knopman et al. analyzed data from the multinational ADCS trial of tocopherol and selegiline, comparing people with AD dementia who moved to a nursing home (*n* = 114) with those who did not (*n* = 227) (81). While baseline CDR-SB scores were not linked to nursing home placement, there was a significant link between reaching CDR 3.0 over 2 years’ follow-up, versus not reaching CDR 3.0, and nursing home placement (OR adjusted for baseline Mini Mental State Examination [MMSE] and total Behavior Rating Scale for Dementia scores, 7.0; 95% CI, 3.99–12.37; HR adjusted for baseline MMSE, 4.8; *p* < 0.001) (81). Ota et al. analyzed data from 633 people with MCI (etiology not specified) or AD dementia in Japan (82). Baseline CDR score was linked to the risk of nursing home placement during follow-up, although the duration of follow-up was not stated. Relative to a CDR score of 0.5, HRs were 1.40 (95% CI, 0.78–2.53; *p* = 0.269) for CDR 1.0, 2.82 (1.52–5.22; *p* = 0.001) for CDR 2.0, and 6.13 (2.47–15.24; *p* < 0.000) for CDR 3.0 (82). However, Rountree et al. followed 500 people with AD dementia (country not reported) for 20 years, and found that change in CDR-SB score was not related to the time to nursing home placement (84).

One study included only cross-sectional data. Ton et al. analyzed secondary data from 121 people with amnestic MCI and 174 with mild, moderate, or severe AD in the US Ageing, Demographics and Memory Study (ADAMS), with normal cognition as the reference group. Higher baseline disease severity, as determined via CDR-SB score category, was associated with use of nursing homes during the preceding 2 years (OR from linear trend tests across all five cognitive states, 2.28; 95% CI, 1.64–3.17; *p* < 0.001) (68).

### 3.7. Costs and healthcare resource use

Six studies reported data on costs and/or healthcare resource use (68, 85–89) (Supplementary Table 4). These data clearly demonstrated a strong relationship between the increasing requirement for formal and informal care with AD progression as assessed by CDR/CDR-SB scores. Direct medical costs also increased in the later stages of AD and were linked to the cost of residential care.

Two analyses used data from the Alzheimer’s disease Follow-Up Study (ALSOVA) study in Finland. Ruokostenpohja et al. assessed the likelihood of receiving the Finnish care partner’ allowance (89), which is a fee paid to family care partners providing care at home to a person with reduced functional capacity, illness, or disability. The allowance is not an automatic entitlement, and the value varies according to region (91). CDR-SB score was significantly associated with the likelihood of receiving the care partners’ allowance (89). Declining ADCS-ADL score was also a significant predictor of receiving the care partners’ allowance, but increasing NPI score was not a predictor (89). Jetsonen et al. found that people in higher baseline CDR-SB score categories incurred significantly higher annual costs for both formal and informal care during 5 years’ of follow-up, relative to those with lower CDR-SB scores (88).

In the analysis of data from the US ADAMS study reported by Ton et al. individuals in higher CDR-SB score categories had a relatively higher chance of using home care, as well as higher direct medical costs and lower household income (68). In an analysis of baseline multinational RCT data by Gustavsson et al., CDR-SB score was correlated with the following cost categories: total informal care (value of lost productivity for care partners younger than 65 years, and value of lost leisure time for those 65 years or older, not including time spent on supervision), patient accommodation, and community services (86). In this study, declining ADCS-ADL score was also correlated with these cost categories, and increasing total NPI score was correlated with use of informal care and community services. ADLs were identified as the largest cost driver in the analyses, with a 1-point decrease in ADCS-ADL score translating into a 3.6% increase in total costs of care (86).

**Figure 4:**
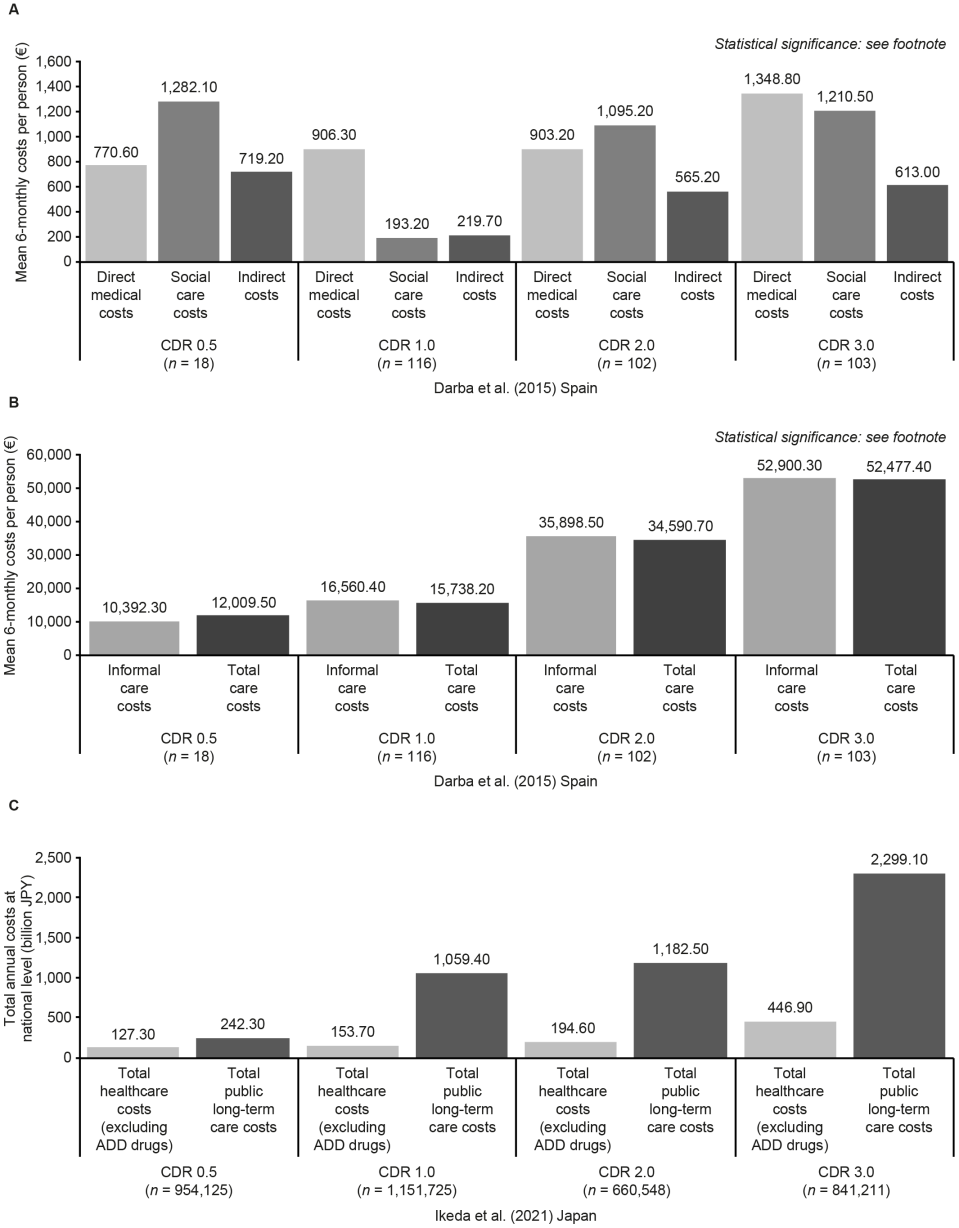
Economic costs associated with CDR score categories in Darba et al. (85) **(A,B)** and Ikeda et al. (87) **(C)**. Statistical significance in Darba et al. (85). Direct medical costs: *p* = 0.02 (CDR 1.0 and CDR 3.0). Social care costs *p* < 0.001 (CDR 1.0 and CDR 2.0; CDR 1.0 and CDR 3.0). Indirect costs: *p* = 0.9. Informal care costs and total care costs: *p* < 0.001 (CDR 0.5 and CDR 2.0, CDR 0.5 and CDR 3.0, CDR 1.0 and CDR 2.0, CDR 1.0 and CDR 3.0, and CDR 2.0 and CDR 3.0). Sample sizes in Ikeda et al. (87) refer to estimated patient numbers for the whole of Japan. Statistical significance was not tested. Estimated per-person costs are also included in the study publication but are not presented here because an estimate for total per-person healthcare costs for CDR 0.5 was not available. ADD, Alzheimer’s disease dementia; CDR, Clinical Dementia Rating; JPY, Japanese Yen.

Two studies reported cross-sectional data by CDR score. Darba et al. found that direct medical care costs increased with CDR score in a Spanish population, particularly at CDR 3.0. There were also notable increases in informal care costs (contributed by time spent on ADLs, IADLs and supervision) at CDR 2.0 and 3.0, compared with earlier stages. Social care costs and indirect costs arising from lost productivity of the care partner were highest at CDR 0.5, and lowest at CDR 1.0 (Figures 4A,B) (85). Ikeda et al. conducted a targeted literature review in Japan, finding that higher public long-term care costs and total medical costs (excluding AD medications) were linked to higher CDR scores (Figure 4C) (87).

Data on resource use were mixed. Gustavsson et al. found no significant association between CDR-SB score and hospitalization (86); however Ton et al. reported that increasing CDR-SB score was associated with a higher likelihood of hospitalization but a decreasing chance of outpatient visits (68). There was a linear but less pronounced relationship between increasing CDR-SB score and visits to a doctor and the same was true of drug utilization (68).

### 3.8. Summary of outcomes by CDR score

Figures 2–4 and Table 4 summarize the 12 studies that reported outcomes by CDR score category (37, 41, 49, 53, 54, 57, 62, 65, 72, 82, 85, 87). These studies suggest that NPI score increases with progression from MCI due to AD to mild AD dementia (CDR 0.5 to CDR 1.0) (53, 54). Both the number of NPS and the frequency of specific NPS increased across CDR score categories (37, 41, 57, 62, 65, 72). Caputo et al. found that hallucinations, and apathy were significantly more common at CDR 2.0 and CDR 3.0 than at CDR 1.0, and disinhibition and aberrant motor behavior were significantly more common at CDR 3.0 than at CDR 1.0 (37). Most CVD comorbidities had no clear relationship with CDR score, but people with a score of CDR 2.0 were slightly more likely to have experienced previous hypertension or heart disease than those at CDR 0.5 or CDR 1.0 (50). A steady decline in ADL scores was observed across CDR score categories, including between CDR 0.5 and CDR 1.0 (49, 53, 62). The likelihood of nursing home placement increased across CDR score categories (82). Accordingly, both Darba et al. (85) and Ikeda et al. (87) found that care costs increased with CDR score, with the largest increases being seen at CDR 3.0. However, there was no clear linear relationship between CDR score and healthcare costs in these studies. Darba et al. found that direct medical costs were higher at CDR 0.5 than at CDR 1.0, and increased again at CDR 2.0 and CDR 3.0, and in Ikeda et al. total healthcare costs did not increase notably until CDR 3.0.

**Table 4:** Outcomes by CDR score.

Study and country	Population	Outcome/measure	Baseline CDR score^a^				*p* value
			0.5	1.0	2.0	3.0	
NPI score							
Hallikainen et al. (53) Finland	*N* = 115 with AD	NPI score, mean (SEM)	Baseline: 6.1 (0.9)	Baseline: 10.2 (1.2)	NR	NR	*p =* 0.005
			1 year: 7.7 (1.1)	1 year: 10.6 (1.4)			*p* = 0.057
			2 years: 9.7 (1.2)	2 years: 14.4 (1.7)			*p* = 0.044
			3 years: 11.6 (1.6)	3 years: 16.6 (1.7)			*p* = 0.006
Hallikainen et al. (54) Finland	*N* = 236 with AD	NPI score, mean (SD)	Baseline: 7.7 (8.3)	Baseline: 10.3 (11.0)	NR	NR	NR
			1 year: 9.6 (10.0)	1 year: 14.0 (13.8)			
			2 years: 11.3 (11.4)	2 years: 17.2 (14.4)			
			3 years: 12.2 (13.2)	3 years: 18.7 (13.1)			
			4 years: 13.4 (12.4)	4 years: 23.0 (17.8)			
			5 years: 13.3 (11.9)	5 years: 22.5 (16.5)			
Naurhashemi et al. (62) France	*N* = 682 with AD	NPI score, mean (SD)	Baseline: 11.2 (12.9)	Baseline: 11.5 (16.0)			*p* < 0.0001
Number of NPS							
Charernboon et al. (41) (Thailand)	*N* = 62 with AD dementia	Number of NPS, mean (SD)	NR	4.3 (1.9)	6.4 (2.2)	7.3 (2.8)	*p* < 0.001
Specific NPS							
Naurhashemi et al. (62) France	*N* = 682 with AD	Frequency of NPS, %	See Figure 2	See Figure 2; NPS frequency was generally higher for CDR ≥ 1.0 than for CDR 0.5			See Figure 2
Kabeshita et al. (57) Japan	*N* = 684 with AD	Frequency of NPS, %	See Figure 2; patterns varied by specific NPS				NR
Caputo et al. (37) Italy	*N* = 690 with AD dementia	Frequency of NPS, %	NR	See Figure 2; patterns varied by specific NPS			
		Association between clinically relevant NPS (frequency × severity ≥4) vs. CDR 1.0, OR (95% CI)		Ref.	Hallucinations: 3.0 (1.3–7.3) Depression: 1.5 (1.1–2.2) Apathy: 1.8 (1.2–2.6)	Delusions: 1.9 (1.1–3.3) Hallucinations: 4.8 (1.9–12.1) Apathy: 1.7 (1.1–2.7) Disinhibition: 3.4 (1.5–7.8) Aberrant motor behavior: 3.5 (2.1–5.9)	NA
Royall et al. (65) USA	*N* = 70 with MCI due to AD or AD dementia	GDS score (subject-rated), mean (SD)	1.9 (1.6)	1.7 (1.4)	NR	NR	NR
		GDS score (care partner-rated), mean (SD)	3.2 (2.8)	4.0 (3.3)			
		Self-reported history of depression, %	29.8	34.8			
Yang et al. (72) USA	*n* = 203 with AD	Frequency of depression at baseline, *n* (%)	48 (37.5)	70 (44.9)	NR	NR	NR
	*n* = 158 with AD and arteriolosclerosis		28 (26.2)	36 (30.0)			
CVD and cardiovascular risk factors							
Eldholm et al. (49) Norway	*N* = 282 with very mild, mild, or moderate AD dementia	Frequency of comorbidities at baseline, %	See Figure 3; patterns varied by specific comorbidity			NR	See Figure 3
Yang et al. (72) USA	*n* = 203 with AD *n* = 158 with AD and arteriolosclerosis	Frequency of comorbidities at baseline, %	See Figure 3; no clear patterns observed		NR	NR	NR
ADLs							
Naurhashemi et al. (62) France	*N =* 682 with AD	ADL score, mean (SD)	5.85 (0.4)	5.22 (1.01)			*p* < 0.0001
		IADL score, mean (SD)	6.04 (1.67)	3.40 (1.91)			*p* < 0.0001
Hallikainen et al. (53) Finland	*N* = 115 with AD	ADCS-ADL score, mean (SEM)	Baseline: 70.3 (0.6)	Baseline: 62.0 (1.1)	NR	NR	*p* < 0.001
			1 year: 66.1 (0.9)	1 year: 55.3 (1.6)			*p* < 0.001
			2 years: 60.2 (1.4)	2 years: 50.0 (1.9)			*p* < 0.001
			3 years: 54.0 (1.9)	3 years: 40.6 (2.3)			*p* < 0.001
Eldholm et al. (49) Norway	*N* = 282 with very mild, mild, or moderate AD dementia	IADL score, mean (SD)	0.90 (0.15)	0.69 (0.20)	0.45 (0.22)	NR	*p* < 0.001
Nursing home placement							
Ota et al. (82) Japan	*N* = 633 with MCI or AD dementia	Individuals who had experienced nursing home placement by the end of follow-up, *n* (%)	107 (64.1)	181 (68.8)	155 (85.6)	22 (100)	NR
		Cox HR (95% CI) for risk of placement, relative to CDR 0.5 in multivariate analysis	Ref.	1.40 (0.78–2.53)	2.82 (1.52–5.22)	6.13 (2.47–15.24)	CDR 1.0: *p* = 0.269 CDR 2.0: *p* = 0.001 CDR 3.0: *p* < 0.000
Economic outcomes							
Darba et al. (85) Spain	*N* = 343 with AD	Direct medical, social care, indirect (lost productivity of care partner), and care costs	See Figure 4; direct medical costs and care costs increased with CDR score; patterns were more complex for other costs				NR
Ikeda et al. (87) Japan	*N* = 3.6 million with AD (estimated population with AD in Japan)	Total healthcare costs and total public long-term care costs	See Figure 4; both types of costs increased with CDR score				NR

AD, Alzheimer’s disease; ADCS-ADL, Alzheimer’s Disease Cooperative Study–Activities of Daily Living scale; ADLs, activities of daily living; CDR, Clinical Dementia Rating; CI, confidence interval; CVD, cardiovascular disease; GDS, Global Deterioration Scale; HR, hazard ratio; IADL, instrumental activities of daily living; MCI, mild cognitive impairment; NA, not applicable; NPI, Neuropsychiatric Inventory; NPS, neuropsychiatric symptom(s); NR, not reported; Ref., reference; SD, standard deviation; SEM, standard error of the mean.

a CDR score categories indicate score at baseline, except where other time points are indicated.

## 4. Discussion

In this comprehensive SLR, we included a broad range of outcomes. We found strong evidence for a link between CDR or CDR-SB scores and NPS, although there was variation across the studies in terms of the specific symptoms linked to AD severity or progression. The relationships between AD progression and CVD were highly inconsistent across studies: although there was some evidence for an association between individual cardiovascular conditions, such as stroke, or risk factors such as diabetes, and CDR or CDR-SB scores, other studies found no such links. There were strong correlations between CDR-SB or CDR scores and a wide range of different ADL measures, indicating that the functional measures included in the CDR scale align closely with ability to perform ADLs. This may also be explained by close overlap between the categories included in the CDR scale and the functional categories in many ADL scales. Multiple studies found that both NPS and ADLs worsen even in the early stages of AD, with decline apparent from CDR 0.5 (MCI due to AD) and CDR 1.0 (mild AD dementia).

Nursing home placement was linked to AD severity and progression, although not all studies found significant correlations, and the study with the longest time horizon found no association between CDR-SB score change and time to placement (84). The use of nursing homes is likely to be particularly dependent on multiple factors that differ across studies and may be difficult to control for, such as the involvement of care partners (92), cultural, ethnic or socioeconomic disparities (93, 94), and the nature and extent of formal care available (95). Therefore, likelihood and timing of placement may differ between countries and geographical regions. However, economic data from Europe, Asia, and the USA indicated that higher CDR-SB or CDR scores are linked to increased requirements for both formal and informal care in all regions, with a stepwise progression of costs across AD stages and a large increase observed at CDR 3.0. Increasing AD severity was also linked to reductions in household income in one study (68), highlighting the broader impact of AD on affected individuals and their care partners. Declining ability to perform ADLs was identified as a driver of care requirements in two studies (86, 89). The first of these two studies found that increasing NPI scores can drive care costs, although less so than declining ADCS-ADL scores (86), but the second study found that NPI score did not predict receipt of a care partners’ allowance (89). A relationship between NPI score and the requirement for care is well recognized, and higher NPI scores have been linked to increases in unpaid care, long-term care, prescription medication use, and physician visits (96). However, the impacts of specific NPS on care requirements and costs differ: an analysis using data from the Cache County Dementia Progression Study found that aggression, psychotic symptoms (delusions and hallucinations), and affective symptoms (depression, anxiety, and irritability) had greater impacts on informal care costs than apathy, sleep disturbances, or appetite disturbances (97).

Direct medical costs typically increased with increasing CDR-SB or CDR score in the studies identified in this SLR, but this was most apparent at later stages of AD dementia. This can be contrasted with the results of a recent analysis of US data, in which the costs of unpaid care increased steadily with AD stage, but direct medical costs, which were assumed to be identical for people receiving care at home and those receiving care in an institution, were similar for all stages of AD (98).

With the exception of some comorbidities, the SLR identified a number of relevant studies for each outcome of interest. There was also good geographical coverage and representation of Asia, Europe, and North America in the studies identified, although other regions, notably South America, Africa, and the Middle East, were not well represented in the data. Some countries were over-represented within particular regions, such as wealthier Asian countries (South Korea and Japan), and Nordic countries with ongoing cohort studies that have generated multiple publications, such as ALSOVA in Finland and the Progression of AD and Resource use (PADR) study in Norway. Disparities among study populations and designs, particularly in terms of demographic factors, AD dementia stage and/or adjustment for these factors, mean that the collation of data should be carried out with caution. For example, studies focused only on patients with a care partner will not be representative of patients with AD living in different circumstances, and the subsets of studies in this SLR that included only patients with MCI due to AD or mild AD dementia may not detect associations between CDR-SB/CDR progression and outcomes that occur predominantly at more severe stages, such as nursing home placement. Economic and healthcare resource utilization data should be interpreted in the context of the healthcare system in each relevant country. As the majority of studies did not confirm AD diagnosis via the assessment of biomarkers, it is highly likely that some patients did not have AD, which may have introduced bias into the findings.

The design of the SLR required that there must be an indication in the title or abstract that relevant outcomes are reported alongside CDR-SB or CDR score. Therefore, it is likely that some excluded publications reported relevant data as baseline characteristics, which were not identified. Comorbidity search terms in the SLR were not exhaustive, and there are additional outcomes that may be relevant to AD, such as cancer, weight loss, and use of antidementia or psychotropic medications, that were not included as search terms. Our searches primarily identified studies that used formal NPS and ADL scales, and more qualitative assessments of factors that are important to patients and care partners, such as the US What Matters Most survey, which highlighted memory and communication problems as key issues (99), were not included in the review.

In conclusion, NPS, ADLs, and costs of care are clearly linked to AD severity and progression, as measured using CDR or CDR-SB scores, beginning at the earliest stages of AD. Although data are available on the links between cardiovascular comorbidities and AD progression, the results of published studies are not consistent, and further investigation is warranted. Future studies on nursing home placement, healthcare costs and resource use, and the relationships between these outcomes, ADLs and NPI, ideally using biomarkers to confirm AD diagnosis, would be highly valuable to indicate how the effects of AD on patients, care partners, and healthcare systems might be mitigated. Further assessments of correlations between rating scales commonly used in AD and dementia should be conducted, to determine what degree of change is clinically meaningful in different populations. Our findings indicate that CDR and CDR-SB scores correlate with multiple patient-and care partner-relevant measures and are a reliable proxy measure for the burden of AD.

## Author contributions

All authors contributed to design of the systematic literature review and data interpretation. CF conducted the systematic literature review. All authors contributed to review and revision of the manuscript, and approved it for publication.

## Funding

This systematic literature review was funded by Novo Nordisk A/S. Medical writing support was provided by Oxford PharmaGenesis, Oxford, UK with funding from Novo Nordisk A/S.

## Conflict of interest

KL has acted as an advisor/consultant for BioXcel Therapeutics, Bright Minds Biosciences, Cerevel Therapeutics, Eisai, GW Pharmaceuticals, IGCPharma, Kondor Pharma, Lundbeck, Merck, Novo Nordisk A/S, Praxis Precision Medicines, and Sumitomo Pharma. JH-P, AC, and LT are employed by Novo Nordisk A/S, which funded the systematic literature review. They contributed to design of the systematic literature review, data interpretation, review and revision of the manuscript, and approval for publication. CE and CF are employed by Oxford PharmaGenesis, which received funding for conducting the systematic literature review. JC has provided consultation to Acadia, Alkahest, AlphaCognition, AriBio, Biogen, Cassava, Cortexyme, Diadem, EIP Pharma, Eisai, GemVax, Genentech, Green Valley, Grifols, Janssen, Karuna, Lilly, LSP, Merck, NervGen, Novo Nordisk A/S, Oligomerix, Ono, Otsuka, PRODEO, Prothena, ReMYND, Resverlogix, Roche, Signant Health, Suven, and United Neuroscience pharmaceutical, assessment, and investment companies. He is supported by US National Institute of General Medical Sciences (NIGMS) grant P20GM109025; National Institute of Neurological Disorders and Stroke (NINDS) grant U01NS093334; National Institute on Aging (NIA) grants R01AG053798, P20AG068053, P30AG072959, and R35AG71476; the Alzheimer’s Disease Drug Discovery Foundation (ADDF); the Ted and Maria Quirk Endowment; and the Joy Chambers-Grundy Endowment.

## Publisher’s note

All claims expressed in this article are solely those of the authors and do not necessarily represent those of their affiliated organizations, or those of the publisher, the editors and the reviewers. Any product that may be evaluated in this article, or claim that may be made by its manufacturer, is not guaranteed or endorsed by the publisher.

## References

[1] Marshall GA Amariglio RE Sperling RA Rentz DM. Activities of daily living: where do they fit in the diagnosis of Alzheimer’s disease? Neurodegener Dis Manag. (2012) 2:483–91. doi: 10.2217/nmt.12.55, PMID: 23585777 PMC3622716

[2] Lyketsos CG Carrillo MC Ryan JM Khachaturian AS Trzepacz P Amatniek J. Neuropsychiatric symptoms in Alzheimer’s disease. Alzheimers Dement. (2011) 7:532–9. doi: 10.1016/j.jalz.2011.05.2410, PMID: 21889116 PMC3299979

[3] Alzheimer’s Disease International. Global estimates of informal care. (2018). Available at: www.alzint.org/u/global-estimates-of-informal-care.pdf [Accessed December 23, 2022].

[4] Toot S Swinson T Devine M Challis D Orrell M. Causes of nursing home placement for older people with dementia: a systematic review and meta-analysis. Int Psychogeriatr. (2017) 29:195–208. doi: 10.1017/S1041610216001654, PMID: 27806743

[5] Kosaner KliessMMartinsRConnollyMP. Major cost drivers in assessing the economic burden of Alzheimer’s disease: a structured, rapid review. J Prev Alzheimers Dis. (2021) 8:362–70. doi: 10.14283/jpad.2021.17, PMID: 34101795

[6] Deb A Thornton JD Sambamoorthi U Innes K. Direct and indirect cost of managing alzheimer’s disease and related dementias in the United States. Expert Rev Pharmacoecon Outcomes Res. (2017) 17:189–202. doi: 10.1080/14737167.2017.1313118, PMID: 28351177 PMC5494694

[7] Wong W. Economic burden of Alzheimer disease and managed care considerations. Am J Manag Care. (2020) 26:S177–83. doi: 10.37765/ajmc.2020.88482, PMID: 32840331

[8] Alzheimer’s Association. 2022 Alzheimer’s Disease Facts and Figures. (2023). Available at: https://www.alz.org/media/Documents/alzheimers-facts-and-figures.pdf [Accessed February 24, 2023].

[9] Kuo TC Zhao Y Weir S Kramer MS Ash AS. Implications of comorbidity on costs for patients with Alzheimer disease. Med Care. (2008) 46:839–46. doi: 10.1097/MLR.0b013e318178940b, PMID: 18665064

[10] Liu YS Barner JC Rascati KL Bhattacharjee S. Economic burden of chronic comorbidities among community-dwelling older adults with dementia: a propensity score matched National-Level Study. Alzheimer Dis Assoc Disord. (2022) 36:244–52. doi: 10.1097/WAD.0000000000000504, PMID: 35293380

[11] Santiago JA Potashkin JA. The impact of disease comorbidities in Alzheimer’s disease. Front Aging Neurosci. (2021) 13:631770. doi: 10.3389/fnagi.2021.631770, PMID: 33643025 PMC7906983

[12] Knight ADRC. CDR® dementia staging instrument. (2015). Available at: https://knightadrc.wustl.edu/professionals-clinicians/cdr-dementia-staging-instrument/ [Accessed December 23, 2022].

[13] Cummings J. The neuropsychiatric inventory: development and applications. J Geriatr Psychiatry Neurol. (2020) 33:73–84. doi: 10.1177/0891988719882102, PMID: 32013737 PMC8505128

[14] Page MJ McKenzie JE Bossuyt PM Boutron I Hoffmann TC Mulrow CD. The PRISMA 2020 statement: an updated guideline for reporting systematic reviews. Syst Rev. (2021) 10:89. doi: 10.1186/s13643-021-01626-4 33781348 PMC8008539

[15] Cummings JL Mega M Gray K Rosenberg-Thompson S Carusi DA Gornbein J. The neuropsychiatric inventory: comprehensive assessment of psychopathology in dementia. Neurology. (1994) 44:2308–14. doi: 10.1212/WNL.44.12.2308, PMID: 7991117

[16] Cummings JL. The neuropsychiatric inventory: assessing psychopathology in dementia patients. Neurology. (1997) 48:S10–6. doi: 10.1212/wnl.48.5_suppl_6.10s 9153155

[17] Katz S. Assessing self-maintenance: activities of daily living, mobility, and instrumental activities of daily living. J Am Geriatr Soc. (1983) 31:721–7. doi: 10.1111/j.1532-5415.1983.tb03391.x 6418786

[18] Wallace M. Katz index of independence in Activities of Daily Living (ADL). (2007). Available at: https://www.alz.org/careplanning/downloads/katz-adl.pdf [Accessed March 10, 2023]. 17390935

[19] Lawton MP Brody EM. Assessment of older people: self-maintaining and instrumental activities of daily living. Gerontologist. (1969) 9:179–86. doi: 10.1093/geront/9.3_Part_1.179, PMID: 5349366

[20] Graf C. The Lawton Instrumental Activities of Daily Living (IADL) scale. (2008). Available at: https://www.alz.org/careplanning/downloads/lawton-iadl.pdf [Accessed March 10, 2023]. 19051984

[21] Park KW Pavlik VN Rountree SD Darby EJ Doody RS. Is functional decline necessary for a diagnosis of Alzheimer’s disease? Dement Geriatr Cogn Disord. (2007) 24:375–9. doi: 10.1159/000109268, PMID: 17914262

[22] Physical Self-Maintenance Scale (n.d.). Physical Self-Maintenance Scale (Activities of Daily Living, or ADLs). Available at: https://www.psychdb.com/_media/teaching/psms.pdf [Accessed March 10, 2023].

[23] Galasko D Bennett D Sano M Ernesto C Thomas R Grundman M. An inventory to assess activities of daily living for clinical trials in Alzheimer’s disease. The Alzheimer’s disease cooperative study. Alzheimer Dis Assoc Disord. (1997) 2:S33–9. doi: 10.1097/00002093-199700112-00005 9236950

[24] Kahle-Wrobleski K Coley N Lepage B Cantet C Vellas B Andrieu S. Understanding the complexities of functional ability in Alzheimer’s disease: more than just basic and instrumental factors. Curr Alzheimer Res. (2014) 11:357–66. doi: 10.2174/1567205011666140317101419, PMID: 24635843 PMC4021450

[25] Jutten RJ Peeters CFW Leijdesdorff SMJ Visser PJ Maier AB Terwee CB. Detecting functional decline from normal aging to dementia: development and validation of a short version of the Amsterdam IADL questionnaire. Alzheimers Dement (Amst). (2017) 8:26–35. doi: 10.1016/j.dadm.2017.03.002 28462387 PMC5403784

[26] Dubbelman MA Terwee CB Verrijp M Visser LNC Scheltens P Sikkes SAM. Giving meaning to the scores of the Amsterdam instrumental activities of daily living questionnaire: a qualitative study. Health Qual Life Outcomes. (2022) 20:47. doi: 10.1186/s12955-022-01958-2 35331258 PMC8943938

[27] Pfeffer RI Kurosaki TT Harrah CH Jr Chance JM Filos S. Measurement of functional activities in older adults in the community. J Gerontol. (1982) 37:323–9. doi: 10.1093/geronj/37.3.323, PMID: 7069156

[28] Mayo AM. Use of the Functional Activities Questionnaire in older adults with dementia. (2016). Available at: https://www.alz.org/careplanning/downloads/functional-activities-questionnaire.pdf [Accessed March 10, 2023].

[29] Reisberg B. Functional assessment staging (FAST). Psychopharmacol Bull. (1988) 24:653–9. PMID: 3249767

[30] Medical Care Corporation (n.d.). Functional Assessment Staging Test. Available at: https://www.mccare.com/pdf/fast.pdf [Accessed March 10, 2023].

[31] Stern Y Albert SM Sano M Richards M Miller L Folstein M. Assessing patient dependence in Alzheimer’s disease. J Gerontol. (1994) 49:M216–22. doi: 10.1093/geronj/49.5.M216, PMID: 8056940

[32] Zhu CW Bruinsma BG Stern Y. Utility of the dependence scale in dementia: validity, meaningfulness, and health economic considerations. Alzheimers Res Ther. (2018) 10:78. doi: 10.1186/s13195-018-0414-7 30103820 PMC6090802

[33] Bandyopadhyay TK Biswas A Roy A Guin DS Gangopadhyay G Sarkhel S. Neuropsychiatric profiles in patients with Alzheimer’s disease and vascular dementia. Ann Indian Acad Neurol. (2014) 17:325–30. doi: 10.4103/0972-2327.138520, PMID: 25221405 PMC4162022

[34] Barca ML Persson K Eldholm R Benth JS Kersten H Knapskog AB. Trajectories of depressive symptoms and their relationship to the progression of dementia. J Affect Disord. (2017) 222:146–52. doi: 10.1016/j.jad.2017.07.008, PMID: 28704802

[35] Bleckwenn M Kleineidam L Wagner M Jessen F Weyerer S Werle J. Impact of coronary heart disease on cognitive decline in Alzheimer’s disease: a prospective longitudinal cohort study in primary care. Br J Gen Pract. (2017) 67:e111–7. doi: 10.3399/bjgp16X688813, PMID: 27993897 PMC5308117

[36] Breitve MH Hynninen MJ Bronnick K Chwiszczuk LJ Auestad BH Aarsland D. A longitudinal study of anxiety and cognitive decline in dementia with Lewy bodies and Alzheimer’s disease. Alzheimers Res Ther. (2016) 8:3. doi: 10.1186/s13195-016-0171-4 26812908 PMC4729131

[37] Caputo M Monastero R Mariani E Santucci A Mangialasche F Camarda R. Neuropsychiatric symptoms in 921 elderly subjects with dementia: a comparison between vascular and neurodegenerative types. Acta Psychiatr Scand. (2008) 117:455–64. doi: 10.1111/j.1600-0447.2008.01175.x, PMID: 18363771

[38] Caroline C Chan PC. Baseline behavioural symptoms impact on clinical disease progression in Alzheimer’s dementia. Ann Acad Med Singap. (2015) 44:S340.

[39] Castrillo Sanz A Andres Calvo M Repiso Gento I Izquierdo Delgado E Gutierrez Rios R Rodriguez Herrero R. Anosognosia in Alzheimer disease: prevalence, associated factors, and influence on disease progression. Neurologia. (2016) 31:296–304. doi: 10.1016/j.nrl.2015.03.006, PMID: 25976940

[40] Chang CW Juan YS Yang YH Lee HY. The relationship between lower urinary tract symptoms and severity of Alzheimer’s disease. Am J Alzheimers Dis Other Dement. (2021) 36:1533317521992657. doi: 10.1177/1533317521992657, PMID: 33635087 PMC10623918

[41] Charernboon T Phanasathit M. Prevalence of neuropsychiatric symptoms in Alzheimer’s disease: a cross-sectional descriptive study in Thailand. J Med Assoc Thail. (2014) 97:560–5. PMID: 25065098

[42] Chew J Abengana J Ali N Chan M Tay L Lim WS. Self-reported sleep duration as a predictor of cognitive decline in mild cognitive impairment (MCI) and mild Alzheimer’s dementia (AD). Alzheimers Dement. (2019) 15:P1185. doi: 10.1016/j.jalz.2019.06.3589

[43] Chou PS Kao YH Wu MN Chou MC Chen CH Lin RT. Effect of the interaction between hypertension and cerebral white matter changes on the progression of Alzheimer disease. Curr Alzheimer Res. (2018) 15:1354–60. doi: 10.2174/1567205015666181002141013, PMID: 30277152

[44] D’Onofrio G Sancarlo D Panza F Copetti M Cascavilla L Paris F. Neuropsychiatric symptoms and functional status in Alzheimer’s disease and vascular dementia patients. Curr Alzheimer Res. (2012) 9:759–71. doi: 10.2174/156720512801322582, PMID: 22715983

[45] de Oliveira FF Chen ES Smith MC Bertolucci PH. Associations of blood pressure with functional and cognitive changes in patients with Alzheimer’s disease. Dement Geriatr Cogn Disord. (2016) 41:314–23. doi: 10.1159/000447585, PMID: 27398980

[46] de Oliveira FF Chen ES Smith MC Bertolucci PHF. Longitudinal lipid profile variations and clinical change in Alzheimer’s disease dementia. Neurosci Lett. (2017) 646:36–42. doi: 10.1016/j.neulet.2017.03.003, PMID: 28274859

[47] de Oliveira FF Pereira FV Pivi GAK Smith MC Bertolucci PHF. Effects of APOE haplotypes and measures of cardiovascular risk over gender-dependent cognitive and functional changes in one year in Alzheimer’s disease. Int J Neurosci. (2018) 128:472–6. doi: 10.1080/00207454.2017.1396986, PMID: 29064737

[48] Dost FS Erken N Ontan MS Ates Bulut E Kaya D Kocyigit SE. Muscle strength seems to be related to the functional status and severity of dementia in older adults with Alzheimer’s disease. Curr Aging Sci. (2022) 16:75–83. doi: 10.2174/1573411018666220616114641 35726809

[49] Eldholm RS Barca ML Persson K Knapskog AB Kersten H Engedal K. Progression of Alzheimer’s disease: a longitudinal study in Norwegian memory clinics. J Alzheimers Dis. (2018) 61:1221–32. doi: 10.3233/JAD-170436, PMID: 29254085

[50] Eldholm RS Persson K Barca ML Knapskog AB Cavallin L Engedal K. Association between vascular comorbidity and progression of Alzheimer’s disease: a two-year observational study in Norwegian memory clinics. BMC Geriatr. (2018) 18:120. doi: 10.1186/s12877-018-0813-4 29788900 PMC5964736

[51] Gilbert M Hess K Corcoran C Snyder C Norton M Rao V. Does the length of time between traumatic brain injury and the onset of Alzheimer’s disease affect the rate of cognitive and functional progression?: the Cache County dementia progression study. Alzheimers Dement. (2012) 8:P500–1. doi: 10.1016/j.jalz.2012.05.1357

[52] Gilbert M Snyder C Corcoran C Norton MC Lyketsos CG Tschanz JT. The association of traumatic brain injury with rate of progression of cognitive and functional impairment in a population-based cohort of Alzheimer’s disease: the Cache County dementia progression study. Int Psychogeriatr. (2014) 26:1593–601. doi: 10.1017/S1041610214000842, PMID: 24831798 PMC4180497

[53] Hallikainen I Hanninen T Fraunberg M Hongisto K Valimaki T Hiltunen A. Progression of Alzheimer’s disease during a three-year follow-up using the CERAD-NB total score: Kuopio ALSOVA study. Int Psychogeriatr. (2013) 25:1335–44. doi: 10.1017/S1041610213000653, PMID: 23676340

[54] Hallikainen I Hongisto K Valimaki T Hanninen T Martikainen J Koivisto AM. The progression of neuropsychiatric symptoms in Alzheimer’s disease during a five-year follow-up: Kuopio ALSOVA study. J Alzheimers Dis. (2018) 61:1367–76. doi: 10.3233/JAD-170697, PMID: 29376861

[55] Irimata KE Dugger BN Wilson JR. Impact of the presence of select cardiovascular risk factors on cognitive changes among dementia subtypes. Curr Alzheimer Res. (2018) 15:1032–44. doi: 10.2174/1567205015666180702105119, PMID: 29962344 PMC6162109

[56] Jenner C Reali G Puopolo M Silveri MC. Can cognitive and behavioural disorders differentiate frontal variant-frontotemporal dementia from Alzheimer’s disease at early stages? Behav Neurol. (2006) 17:89–95. doi: 10.1155/2006/812760, PMID: 16873919 PMC5471534

[57] Kabeshita Y Adachi H Matsushita M Kanemoto H Sato S Suzuki Y. Sleep disturbances are key symptoms of very early stage Alzheimer disease with behavioral and psychological symptoms: a Japan multi-center cross-sectional study (J-BIRD). Int J Geriatr Psychiatry. (2017) 32:222–30. doi: 10.1002/gps.4470, PMID: 27001907

[58] Lee WJ Liao YC Wang YF Lin YS Wang SJ Fuh JL. Summative effects of vascular risk factors on the progression of Alzheimer disease. J Am Geriatr Soc. (2020) 68:129–36. doi: 10.1111/jgs.16181 31587263

[59] Mielke MM Rosenberg PB Tschanz J Cook L Corcoran C Hayden KM. Vascular factors predict rate of progression in Alzheimer disease. Neurology. (2007) 69:1850–8. doi: 10.1212/01.wnl.0000279520.59792.fe, PMID: 17984453

[60] Moon Y Moon WJ Kim JO Kwon KJ Joung J Han SH. Predictors of poor clinical outcome and role of muscle profile in Alzheimer’s disease: a 3-year longitudinal study. Alzheimers Dement. (2019) 15:P700–1. doi: 10.1016/j.jalz.2019.06.2705 31655799

[61] Na HR Park MH Cho ST Lee BC Park S Kim KH. Urinary incontinence in Alzheimer’s disease is associated with clinical dementia rating-sum of boxes and Barthel activities of daily living. Asia Pac Psychiatry. (2015) 7:113–20. doi: 10.1111/appy.12007, PMID: 23857871

[62] Naurhashemi F Ousset PJ Gillette-Guyonnet S Cantent C Andrieu S Vellas B. A 2-year follow-up of 233 very mild (CDR 0.5) Alzheimer’s disease patients (REAL.FR cohort). Int J Geriatr Psychiatry. (2008) 23:460–5. doi: 10.1002/gps.1904, PMID: 17894422

[63] Park MH Na H. Urinary incontinence in Alzheimer’s disease is associated with clinical dementia rating: sum of boxes and Barthel’s activities of daily living. Alzheimers Dement. (2012) 8:P128. doi: 10.1016/j.jalz.2012.05.339 23857871

[64] Pavlik VN Chan W Darby E. Cohort effects in progression rate on cognitive and functional measures in an Alzheimer’s disease clinical cohort. J Alzheimers Dis. (2019) 71:659–69. doi: 10.3233/JAD-190661, PMID: 31424408

[65] Royall DR Palmer RF Vidoni ED Honea RA. The default mode network may be the key substrate of depressive symptom-related cognitive changes. Adv Alzheimer’s Dis. (2015) 4:247–61. doi: 10.3233/978-1-61499-542-5-247 PMC3640836 23254633

[66] Shim YS Choi SH Park KW. Response to medical treatment is affected by which neuropsychiatric symptoms patients with Alzheimer’s disease have. Alzheimers Dement. (2015) 11:P671. doi: 10.1016/j.jalz.2015.06.997

[67] Tay L Leung B Yeo A Chan M Lim WS. Elevations in serum Dickkopf-1 and disease progression in community-dwelling older adults with mild cognitive impairment and mild-to-moderate Alzheimer’s disease. Front Aging Neurosci. (2019) 11:278. doi: 10.3389/fnagi.2019.00278 31680933 PMC6803458

[68] Ton TGN DeLeire T May SG Hou N Tebeka MG Chen E. The financial burden and health care utilization patterns associated with amnestic mild cognitive impairment. Alzheimers Dement. (2017) 13:217–24. doi: 10.1016/j.jalz.2016.08.009 27693186

[69] Tschanz JT Corcoran CD Schwartz S Treiber K Green RC Norton MC. Progression of cognitive, functional, and neuropsychiatric symptom domains in a population cohort with Alzheimer dementia: the Cache County dementia progression study. Am J Geriatr Psychiatry. (2011) 19:532–42. doi: 10.1097/JGP.0b013e3181faec23, PMID: 21606896 PMC3101372

[70] Voglein J Ricard I Noachtar S Kukull WA Dieterich M Levin J. Epilepsy in Alzheimer disease is frequent and characterized by high recurrence. Alzheimers Dement. (2019) 15:P700. doi: 10.1016/j.jalz.2019.06.2704

[71] Wadsworth LP Rentz D Lorius N Johnson K Sperling R Locascio J. Neuropsychiatric symptoms are associated with current and future global functional impairment in mild cognitive impairment. Alzheimers Dement. (2011) 7:S168–9. doi: 10.1016/j.jalz.2011.05.456

[72] Yang D Masurkar AV. Clinical profiles of arteriolosclerosis and Alzheimer disease at mild cognitive impairment and mild dementia in a national neuropathology cohort. Alzheimer Dis Assoc Disord. (2021) 35:14–22. doi: 10.1097/WAD.0000000000000411, PMID: 32925200 PMC7904566

[73] Yeo A Chong MS Tay L Yap J Chan M. Assessing clinical progression in Alzheimer’s disease (AD) subjects: an alternative pre-progression rate in a Singapore memory clinic population. Ann Acad Med Singap. (2013) 42:S268.

[74] Zhang W Lü Y Yu W. (2022). Neuropsychiatric symptoms in mild cognitive impairment and Alzheimer’s Disease: relation with disease stage, sex, and daily function impairment. Presented at the Alzheimer’s Association International Conference, July 31 to August 4, San Diego, CA, USA.

[75] Dubbelman M Postema M Scheltens P Van Der Flier W Sikkes S. The Amsterdam instrumental activities of daily living questionnaire: validation of a clinically meaningful outcome measure in asymptomatic and early symptomatic Alzheimer’s disease. J Prev Alzheimers Dis. (2021) 8:S134–5. doi: 10.14283/jpad.2021.58

[76] Hotta M Hashimoto M Fukuhara R Koyama A Murata M Yoshiura K. Relationship between cognitive declines and independency in the activities of daily living in patients with frontotemporal dementia. J Neurochem. (2016) 138:296. doi: 10.1111/jnc.13692

[77] McDougall F Edgar C Mertes M Delmar P Fontoura P Abi-Saab D. Psychometric properties of the clinical dementia rating - sum of boxes and other cognitive and functional outcomes in a prodromal Alzheimer’s disease population. J Prev Alzheimers Dis. (2021) 8:151–60. doi: 10.14283/jpad.2020.73, PMID: 33569561

[78] Na HR Kim SY Chang YH Park MH Cho ST Han IW. Functional assessment staging (FAST) in Korean patients with Alzheimer’s disease. J Alzheimers Dis. (2010) 22:151–8. doi: 10.3233/JAD-2010-100072, PMID: 20847407

[79] Cohen JT McLaughlin TP Neumann P Mucha L Liu E Grundman M. P1‐212: estimating dependence scale scores based on clinical dementia rating ‐ sum of boxes scores in patients with mild cognitive impairment or mild to moderate Alzheimer’s disease. Alzheimers Dement. (2009) 5:238–9. doi: 10.1016/j.jalz.2009.04.219

[80] Jones RW Lacey L Knapp M Romeo R Sato A Niecko T. Poster session 1, Sunday 9 September. Eur J Neurol. (2012) 19:90–457. doi: 10.1111/j.1468-1331.2012.03888.x

[81] Knopman DS Berg JD Thomas R Grundman M Thal LJ Sano M. Nursing home placement is related to dementia progression: experience from a clinical trial. Neurology. (1999) 52:714–8. doi: 10.1212/WNL.52.4.714, PMID: 10078715

[82] Ota K Arikawa M Ohashi S Azekawa T Matsumoto T. Factors influencing nursing home placement of patients with dementia: a retrospective, single-Centre study in Japan. Psychogeriatrics. (2019) 19:111–6. doi: 10.1111/psyg.12373 30294822

[83] Park DG Lee S Moon YM Na DL Jeong JH Park KW. Predictors of institutionalization in patients with Alzheimer’s disease in South Korea. J Clin Neurol. (2018) 14:191–9. doi: 10.3988/jcn.2018.14.2.191, PMID: 29504294 PMC5897202

[84] Rountree S Chan W Pavlik V Doody R. Factors that influence nursing home placement in an Alzheimer’s disease cohort. Alzheimers Dement. (2012) 8:P375. doi: 10.1016/j.jalz.2012.05.1030 PMC3506931 22594761

[85] Darba J Kaskens L Lacey L. Relationship between global severity of patients with Alzheimer’s disease and costs of care in Spain; results from the co-dependence study in Spain. Eur J Health Econ. (2015) 16:895–905. doi: 10.1007/s10198-014-0642-0, PMID: 25348897

[86] Gustavsson A Cattelin F Jonsson L. Costs of care in a mild-to-moderate Alzheimer clinical trial sample: key resources and their determinants. Alzheimers Dement. (2011) 7:466–73. doi: 10.1016/j.jalz.2010.06.002 21784355

[87] Ikeda S Mimura M Ikeda M Wada-Isoe K Azuma M Inoue S. Economic burden of Alzheimer’s disease dementia in Japan. J Alzheimers Dis. (2021) 81:309–19. doi: 10.3233/JAD-210075, PMID: 33780371 PMC8203238

[88] Jetsonen V Kuvaja-Kollner V Valimaki T Selander T Martikainen J Koivisto AM. Total cost of care increases significantly from early to mild Alzheimer’s disease: 5-year ALSOVA follow-up. Age Ageing. (2021) 50:2116–22. doi: 10.1093/ageing/afab144, PMID: 34255025 PMC8581391

[89] Ruokostenpohja N Valimaki T Martikainen J Hallikainen M Vehvilainen-Julkunen K Koivisto A. Entitlement of carer’s allowance to support home care of persons with Alzheimer’s disease: evaluation of current decision criteria. Eur Geriatr Med. (2018) 9:477–83. doi: 10.1007/s41999-018-0060-4, PMID: 34674484

[90] McKhann G Drachman D Folstein M Katzman R Price D Stadlan EM. Clinical diagnosis of Alzheimer’s disease: report of the NINCDS-ADRDA work group under the auspices of Department of Health and Human Services Task Force on Alzheimer’s disease. Neurology. (1984) 34:939–44. doi: 10.1212/WNL.34.7.939, PMID: 6610841

[91] Linnosmaa I Jokinen S Vilkko A Noro A Siljander E. (2014). Report on the fees and services of informal care support in municipalities in 2012. National Institute for Health and Welfare (THL). Helsinki.

[92] Friedman SM Steinwachs DM Temkin-Greener H Mukamel DB. Informal caregivers and the risk of nursing home admission among individuals enrolled in the program of all-inclusive care for the elderly. Gerontologist. (2006) 46:456–63. doi: 10.1093/geront/46.4.456, PMID: 16920999

[93] Estrada LV Agarwal M Stone PW. Racial/ethnic disparities in nursing home end-of-life care: a systematic review. J Am Med Dir Assoc. (2021) 22:e1. doi: 10.1016/j.jamda.2020.12.005 PMC8128037 33428892

[94] Fitzgerald MH Mullavey-O’Byrne C Clemson L. Families and nursing home placements: a cross-cultural study. J Cross Cult Gerontol. (2001) 16:333–51. doi: 10.1023/A:1014505219291, PMID: 14617978

[95] Coe NB Konetzka RT Berkowitz M Blecker E Van Houtven CH. The effects of home care provider mix on the care recipient: an international, systematic review of articles from 2000 to 2020. Annu Rev Public Health. (2021) 42:483–503. doi: 10.1146/annurev-publhealth-090419-102354, PMID: 33395544 PMC8190630

[96] Murman DL Colenda CC. The economic impact of neuropsychiatric symptoms in Alzheimer’s disease: can drugs ease the burden? PharmacoEconomics. (2005) 23:227–42. doi: 10.2165/00019053-200523030-00004, PMID: 15836005

[97] Rattinger GB Sanders CL Vernon E Schwartz S Behrens S Lyketsos CG. Neuropsychiatric symptoms in patients with dementia and the longitudinal costs of informal care in the Cache County population. Alzheimers Dement (N Y). (2019) 5:81–8. doi: 10.1016/j.trci.2019.01.002 30911601 PMC6416410

[98] Razavi M Herring WL Gillis C Maserejian N Pemberton-Ross P Nejati M. (2022). Economic burden of daily transitions to later stages of AD dementia in the US. Poster LP95. Presented at the 15th Clinical Trials on Alzheimer’s Disease (CTAD) Conference, November 29 to December 2, San Francisco, CA, USA.

[99] DiBenedetti DB Slota C Wronski SL Vradenburg G Comer M Callahan LF. Assessing what matters most to patients with or at risk for Alzheimer’s and care partners: a qualitative study evaluating symptoms, impacts, and outcomes. Alzheimers Res Ther. (2020) 12:90. doi: 10.1186/s13195-020-00659-6, PMID: 32731886 PMC7393916

