# Supplementary material for: Exploring the relationship between patient-relevant outcomes and Alzheimer’s disease progression assessed using the clinical dementia rating scale: a systematic literature review
Source: Front Neurol. 2023 Aug 17;14:1208802. doi: 10.3389/fneur.2023.1208802 (PMC10470645; doi:10.3389/fneur.2023.1208802)
Supplement: Supplementary file 1 [file Data_Sheet_1.pdf]

Supplementary Material

Exploring the relationship between patient-relevant outcomes and Alzheimer's disease progression assessed using the Clinical Dementia Rating scale: a systematic literature review

Jeffrey Cummings[1], Julie Hviid Hahn-Pedersen[2], Christian Stefan Eichinger[3], Caroline Freeman[3], Alice Clark[2], Luis Rafael Solís Tarazona[2], Krista Lanctôt[4]

[1]Chambers-Grundy Center for Transformative Neuroscience, Department of Brain Health, School of Integrated Health Sciences, University of Nevada, Las Vegas, NV, USA

[2]Novo Nordisk A/S, Søborg, Denmark

[3]Oxford PharmaGenesis, Oxford, UK

[4]Hurvitz Brain Sciences Program, Sunnybrook Research Institute; and Department of Psychiatry, University of Toronto, Toronto, ON, Canada

*** Correspondence:**
Jeffrey Cummings
jcummings@cnsinnovations.com

1 Supplementary Methods

Systematic searches were performed on September 13, 2022, using Embase, MEDLINE/Medline In-Process, and the Cochrane Library via Ovid. Search terms (Supplementary Table 1) were designed to detect studies reporting data on relationships between Clinical Dementia Rating-Sum of Boxes (CDR-SB) scores and relevant outcomes in people with mild cognitive impairment due to Alzheimer's disease (AD) or AD dementia. Abstracts from the Alzheimer's Association International Conference, the International Conference on Alzheimer's and Parkinson's Diseases, and the American Academy of Neurology Annual Meeting in 2022 were also searched.

Eligibility criteria for inclusion of studies (Table 1 in the main manuscript) were developed according to the PICOS (population, interventions, comparators, outcomes, and study design) framework. Titles and abstracts were screened by one reviewer to determine whether they met the eligibility criteria. All publications that met the criteria for inclusion were obtained as full articles and reassessed against the eligibility criteria. Detailed data on study design, methods and setting, patient demographics, disease status, and study results were extracted from each included publication into a data extraction table.

2 Supplementary Tables

Supplementary Table 1 Electronic search strings for the SLR.

**Embase**		
**#**	**Searches**	**Results**
1	Alzheimer disease/	230099
2	Alzheimer*.ti,ab.	231075
3	1 or 2	276204
4	clinical dementia rating.ti,ab.	4368
5	boxes.ti,ab.	14640
6	4 and 5	913
7	(CDR-SB or CDR SB or CDR score).ti,ab.	1031
8	6 or 7	1560
9	3 and 8	1253
10	mental disease/ or depression/ or epilepsy/ or bedsore/ or comorbidity/ or infection/ or incontinence/ or Cardiovascular Disease/	1701254
11	(psychiatric* or depression* or epileps* or bedsore* or comorbidit* or infection* or incontinence* or cardiovascular*).mp.	5534183
12	((economic or pharmacoeconomic) adj1 (evaluation or assessment or analys?s or stud*)).mp. or Cost effectiveness analysis/ or Cost minimization analysis/ or Cost benefit analysis/ or Cost utility analysis/ or Budget impact/ or Cost consequence analysis/ or (Cost effectiveness analysis or Cost minimization analysis or Cost benefit analysis or Cost utility analysis or Budget impact or Cost consequence analysis).mp. or (CEA or CMA or CBA or CUA or CCA).mp.	361426
13	"health care cost"/ or "drug cost"/ or "hospital cost"/ or "hospitalization cost"/ or "nursing cost"/ or ((health or global) adj2 burden).mp. or ((direct or indirect or societ* or employe*) adj2 (resource* or benefit*)).mp. or exp caregiver burden/ or exp caregiver support/ or (caregiver* or carer*).mp. or economics/ or budget*.mp. or cost*.mp. or productivity/ or productivity.mp. or absenteeism.mp. or absenteeism/ or "length of stay"/ or Cost control/ or (fiscal or financ* or funding).mp. or financial management.mp. or financial management/ or health care utilization/ or health care utili*.mp. or health care financing.mp. or health care financing/ or health economics.mp. or health economics/ or (burden adj2 (illness or disease$ or treatment*)).mp. or resource allocation/ or exp resource management/ or budget/ or pharmacoeconomics/ or pharmacoeconomic*.mp. or pay?r.mp. or health care planning.mp. or health care planning/ or (resource adj2 (use* or utili?ation or allocat* or burden or health)).mp. or	2404602

	(economic adj5 (burden or impact)).mp. or cost of illness.mp. or "cost of illness"/ or cost control.mp. or "cost control"/ or Economics, Medical/	
14	(Dependence scale or Severity of Dependence Scale or SDS).mp.	102593
15	exp Activities of Daily Living/ or exp ADL disability/ or exp Daily life activity/ or exp Leisure/ or ((daily life or daily living or instrument* or leisure or extended) adj3 activit*).tw,kw. or (ADL* or BADL* or IADL* or EADL*).tw,kw. or exp Functional Status/ or exp Functional Assessment/ or (function* adj3 (capacity or independen* or status or assess* or abilit*)).tw,kw.	543883
16	fall*.mp.	347213
17	(((nursing home$ or care home$ or long-term care or institution$ or facility) adj5 (place$ or entry or admit$ or admission$)) or institutionalization).tw. or exp Home for the Aged/ or Nursing Home/	83400
18	sleep disordered breathing/ or (sleep apnoea or sleep apnea).mp. or (coronary disease* or myocardial infarction* or heart failure* or cerebrovascular disease* or stroke* or peripheral vascular disease*).mp.	1357661
19	or/10-18	9161950
20	9 and 19	581
21	(animal$ not human$).sh,hw.	4685409
22	20 not 21	575
23	remove duplicates from 22	565

Ovid MEDLINE(R)

#	Searches	Results
1	Alzheimer disease/	112116
2	Alzheimer*.ti,ab.	171250
3	1 or 2	184504
4	clinical dementia rating.ti,ab.	2692
5	boxes.ti,ab.	11952
6	4 and 5	526
7	(CDR-SB or CDR SB or CDR score).ti,ab.	418
8	6 or 7	758
9	3 and 8	600

10	Mental Disorders/ or depression/ or epilepsy/ or bedsore/ or comorbidity/ or infection/ or Urinary Incontinence/ or Cardiovascular Disease/	734778
11	(psychiatric* or depression* or epileps* or bedsore* or comorbidit* or infection* or incontinence* or cardiovascular*).mp.	3886704
12	((economic or pharmacoeconomic) adj1 (evaluation or assessment or analys?s or stud*)).mp. or Cost effectiveness analysis/ or Cost minimization analysis/ or Cost benefit analysis/ or Cost utility analysis/ or Budget impact/ or Cost consequence analysis/ or (Cost effectiveness analysis or Cost minimization analysis or Cost benefit analysis or Cost utility analysis or Budget impact or Cost consequence analysis).mp. or (CEA or CMA or CBA or CUA or CCA).mp.	224346
13	"health care cost"/ or "drug cost"/ or "hospital cost"/ or "hospitalization cost"/ or "nursing cost"/ or ((health or global) adj2 burden).mp. or ((direct or indirect or societ* or employe*) adj2 (resource* or benefit*)).mp. or exp caregiver burden/ or exp caregiver support/ or (caregiver* or carer*).mp. or economics/ or budget*.mp. or cost*.mp. or productivity/ or productivity.mp. or absenteeism.mp. or absenteeism/ or presenteeism.mp. or presenteeism/ or "length of stay"/ or Cost control/ or (fiscal or financ* or funding).mp. or financial management.mp. or financial management/ or health care utilization/ or health care utili*.mp. or health care financing.mp. or health economics.mp. or (burden adj2 (illness or disease$ or treatment*)).mp. or resource allocation/ or resource allocation.mp. or resource management.mp. or budget/ or budget.mp. or pharmacoeconomics/ or pharmacoeconomic*.mp. or pay?r.mp. or health care planning.mp. or (resource adj2 (use* or utili?ation or allocat* or burden or health)).mp. or (economic adj5 (burden or impact)).mp. or cost of illness.mp. or "cost of illness"/ or cost control.mp. or "cost control"/ or Economics, Medical/	1464632
14	(Dependence scale or Severity of Dependence Scale or SDS).mp.	81787
15	exp Activities of Daily Living/ or exp Leisure/ or ((daily life or daily living or instrument* or leisure or extended) adj3 activit*).tw,kw. or (ADL* or BADL* or IADL* or EADL*).tw,kw. or exp Functional Status/ or (function* adj3 (capacity or independen* or status or assess* or abilit*)).tw,kw.	599393
16	fall*.mp.	275162
17	(((nursing home$ or care home$ or long-term care or institution$ or facility) adj5 (place$ or entry or admit$ or admission$)) or institutionalization).tw. or Nursing Home/	24485
18	sleep disordered breathing/ or (sleep apnoea or sleep apnea).mp. or (coronary disease* or myocardial infarction* or heart failure* or cerebrovascular disease* or stroke* or peripheral vascular disease*).mp.	952459
19	or/10-18	6686216
20	9 and 19	289
21	(animal$ not human$).sh,hw.	5000087
22	20 not 21	289

23	remove duplicates from 22	289
**Cochrane (ACP Journal Club, CDSR, CCTR, DARE, CLEED, CLHTA, CLCMR), run on September 6, 2022**		
**#**	**Searches**	**Results**
1	Alzheimer disease/	3921
2	Alzheimer*.ti,ab.	12909
3	1 or 2	13363
4	clinical dementia rating.ti,ab.	811
5	boxes.ti,ab.	886
6	4 and 5	290
7	(CDR-SB or CDR SB or CDR score).ti,ab.	380
8	6 or 7	497
9	3 and 8	423
10	Mental Disorders/ or depression/ or epilepsy/ or bedsore/ or comorbidity/ or infection/ or Urinary Incontinence/ or Cardiovascular Disease/	28929
11	(psychiatric* or depression* or epileps* or bedsore* or comorbidit* or infection* or incontinence* or cardiovascular*).mp.	376929
12	((economic or pharmacoeconomic) adj1 (evaluation or assessment or analys?s or stud*)).mp. or Cost effectiveness analysis/ or Cost minimization analysis/ or Cost benefit analysis/ or Cost utility analysis/ or Budget impact/ or Cost consequence analysis/ or (Cost effectiveness analysis or Cost minimization analysis or Cost benefit analysis or Cost utility analysis or Budget impact or Cost consequence analysis).mp. or (CEA or CMA or CBA or CUA or CCA).mp.	48005
13	"health care cost"/ or "drug cost"/ or "hospital cost"/ or "hospitalization cost"/ or "nursing cost"/ or ((health or global) adj2 burden).mp. or ((direct or indirect or societ* or employe*) adj2 (resource* or benefit*)).mp. or exp caregiver burden/ or exp caregiver support/ or (caregiver* or carer*).mp. or economics/ or budget*.mp. or cost*.mp. or productivity/ or productivity.mp. or absenteeism.mp. or absenteeism/ or presenteeism.mp. or presenteeism/ or "length of stay"/ or Cost control/ or (fiscal or financ* or funding).mp. or financial management.mp. or financial management/ or health care utilization/ or health care utili*.mp. or health care financing.mp. or health economics.mp. or (burden adj2 (illness or disease$ or treatment*)).mp. or resource allocation/ or resource allocation.mp. or resource management.mp. or budget/ or budget.mp. or pharmacoeconomics/ or pharmacoeconomic*.mp. or pay?r.mp. or health care planning.mp. or (resource adj2 (use* or utili?ation or allocat* or burden or health)).mp. or (economic adj5 (burden or impact)).mp. or cost of illness.mp. or "cost of illness"/ or cost control.mp. or "cost control"/ or Economics, Medical/	165948

14	(Dependence scale or Severity of Dependence Scale or SDS).mp.	5229
15	exp Activities of Daily Living/ or exp Leisure/ or ((daily life or daily living or instrument* or leisure or extended) adj3 activit*).tw,kw. or (ADL* or BADL* or IADL* or EADL*).tw,kw. or exp Functional Status/ or (function* adj3 (capacity or independen* or status or assess* or abilit*)).tw,kw.	85521
16	fall*.mp.	29981
17	(((nursing home$ or care home$ or long-term care or institution$ or facility) adj5 (place$ or entry or admit$ or admission$)) or institutionalization).tw. or Nursing Home/	2086
18	sleep disordered breathing/ or (sleep apnoea or sleep apnea).mp. or (coronary disease* or myocardial infarction* or heart failure* or cerebrovascular disease* or stroke* or peripheral vascular disease*).mp.	136828
19	or/10-18	666770
20	9 and 19	278
21	(animal$ not human$).sh,hw.	2308
22	20 not 21	278
23	remove duplicates from 22	272

Supplementary Table 2 Studies reporting NPS data.

Study and country	Population	AD severity/ staging measure	Results
**NPS**			
Barca et al. (2017) (1)<br>Norway	N = 282 (105 with amnestic MCI, 177 with AD dementia)	CDR	Change in CDR score over follow-up (mean [SD])<br>Class 1 (stable low depressive symptom scores on the Cornell Scale for Depression): 2.9 (3.4)<br>Class 2 (high and decreasing depressive symptom scores on the Cornell Scale for Depression): 3.5 (3.9)<br>Class 3 (moderate and increasing depressive symptom scores on the Cornell Scale for Depression): 4.8 (4.2)<br>p value (Kruskal-Wallis): 0.020<br>p value (Mann-Whitney) for C1 vs. C2: 0.458<br>p value (Mann-Whitney) for C1 vs. C3: 0.006<br>p value (Mann-Whitney) for C2 vs. C3: 0.418<br><br>Bivariate and multivariate models for class 3 membership with class 1 as reference (results the same for both models)<br>CDR change: OR (95% CI): 1.14 (1.03–1.25); p = 0.009
Caroline et al. (2015) (2)<br>China	N = 101 with mild/moderate AD dementia<br>n = 94 with follow-up data	CDR-SB	Delusions, depression, anxiety, apathy, and aberrant motor behavior were significantly more frequent among fast progressors[a] rather than slow progressors over 1 year ($p < 0.05$)

Study and country	Population	AD severity/ staging measure	Results
Hallikainen et al. (2018) (3)<br><br>Finland	*N* = 236 with AD<br>(CDR 0.5 or CDR 1.0)	CDR-SB	Regression coefficients (95% CI) for baseline NPS predicting CDR-SB progression over 5 years' follow-up in multivariate analyses<br><br>Delusions: 0.596 (0.166–1.026); $p = 0.007$<br><br>Agitation: 0.203 (–0.082 to 0.489); $p = 0.162$<br><br>Euphoria: –0.561 (–0.736 to –0.385); $p < 0.001$<br><br>Aberrant motor behavior: 0.295 (–0.004 to 0.594); $p = 0.053$<br><br>Regression coefficients (95% CI) for association between NPS and CDR-SB score over 5 years' follow-up in multivariate analyses<br><br>Delusions: 0.108 (0.037–0.178); $p = 0.003$<br><br>Hallucinations: 0.225 (0.123–0.327); $p < 0.000$<br><br>Agitation: 0.097 (0.001–0.192); $p = 0.047$<br><br>Apathy: 0.178 (0.117–0.240); $p < 0.000$<br><br>Aberrant motor behavior: 0.119 (0.059–0.179); $p < 0.000$<br><br>Sleep disturbances: 0.087 (0.019–0.155); $p = 0.012$<br><br>Appetite disturbances: 0.047 (0.001–0.093); $p = 0.046$
Wadsworth et al. (2011) (4)<br><br>USA	*N* = 583 (395 with MCI and 188 with mild AD)	CDR-SB	In AD, greater baseline CDR-SB was associated with greater apathy on the NPI ($R^2 = 0.17$; $\beta = 0.55$; $p < 0.001$).<br><br>This association was not significant for 2-year CDR-SB change
Breitve et al. (2016) (5)<br><br>Norway	*N* =122 with AD dementia	CDR and CDR-SB	Neither care partner- nor patient-reported anxiety were correlated with cognitive decline or disease severity measured by CDR-SB or CDR (no data reported)

**Study and country**	**Population**	**AD severity/ staging measure**	**Results**
**NPI score**			
Hallikainen et al. (2013) (6)<br><br>Finland	*N* = 115 with AD (CDR 0.5 or CDR 1.0)	CDR and CDR-SB	NPI score, mean (SEM)<br>Baseline<br>CDR 0.5: 6.1 (0.9)<br>CDR 1.0: 10.2 (1.2)<br>$p = 0.005$<br><br>1 year<br>CDR 0.5: 7.7 (1.1)<br>CDR 1.0: 10.6 (1.4)<br>$p = 0.057$<br><br>2 years<br>CDR 0.5: 9.7 (1.2)<br>CDR 1.0: 14.4 (1.7)<br>$p = 0.044$<br><br>3 years<br>CDR 0.5: 11.6 (1.6)<br>CDR 1.0: 16.6 (1.7)<br>$p = 0.006$<br>Correlation between CDR-SB score and NPI score after 3 years: 0.453 ($p < 0.000$)

Study and country	Population	AD severity/ staging measure	Results
Naurhashemi et al. (2008) (7)<br>France	*N* = 682 with AD (CDR 0.5–3.0)	CDR	NPI score, mean (SD)<br>CDR 0.5: 11.18 (12.87)<br>CDR ≥ 1.0: 11.54 (16.02)<br>$p < 0.0001$<br><br>Rate of NPI score progression over follow-up<br>CDR 0.5: +4.24 (15.39)<br>CDR ≥ 1.0: +3.34 (17.10)<br>$p = 0.7939$
Jenner et al. (2006) (8)<br>Italy	*N* = 22 with AD dementia	CDR	Spearman rank order test for CDR vs. NPI: 0.16; *p* = NS
Tschanz et al. (2011) (9)<br>USA	*N* = 328 with AD dementia	CDR-SB	Weak association between the CDR-SB and NPI ($r = 0.20$; df = 206; $p = 0.004$)
Caroline et al. (2015) (2)<br>China	*N* = 101 with mild/moderate AD dementia<br>*n* = 94 with follow-up data	CDR-SB	NPI score was a significant predictor of fast progression[a] in multivariate analyses (OR, 1.26; 95% CI, 1.05–1.51; $p = 0.015$)
Tay et al. (2019) (10)<br>Singapore	*N* = 96 (14 with MCI, 74 with AD dementia)<br>*n* = 88 with follow-up	CDR-SB	NPI score, mean (SD)<br>Baseline<br>Progressors[b]: 5.3 (5.1)<br>Non-progressors: 3.4 (3.3)

**Study and country**	**Population**	**AD severity/ staging measure**	**Results**
			p = 0.198<br><br>1 year<br>Progressors[b]: 7.0 (6.8)<br>Non-progressors: 3.0 (4.0)<br>*p* = 0.042<br><br>Change over 1 year's follow-up<br>Progressors[b]: 1.7 (4.7)<br>Non-progressors: –0.4 (3.2)<br>*p* = 0.033
**Number of NPS**			
Charernboon et al. (2014) (11)<br>Thailand	*N* = 62 with AD dementia	CDR	Number of NPS, mean (SD)<br>CDR 1.0: 4.26 (1.87)<br>CDR 2.0: 6.37 (2.20)<br>CDR 3.0: 7.25 (2.75)<br>The mean number of the CDR 1.0 group was significantly different from the CDR 2.0 and CDR 3.0 groups (*p* = 0.002 and 0.027, respectively), but the CDR 2.0 group was not significantly different from the CDR 3.0 group (*p* = 0.740)
Bandyopadhyay et al. (2014) (12)<br>India	*N* = 50 with AD dementia	CDR	Weak positive correlation between the CDR score and NPS per patient (*r* = 0.217)

[a]Fast progression was defined as a decline of CDR-SB score of ≥ 2 points.

[b]Disease progression defined as an increase of ≥ 2 points from baseline on the CDR-SB.

AD, Alzheimer's disease; CDR, Clinical Dementia Rating; CDR-SB, Clinical Dementia Rating–Sum of Boxes; CI, confidence interval; MCI, mild cognitive impairment; NPI, Neuropsychiatric Inventory; NPS, neuropsychiatric symptom(s); NS, not significant; OR, odds ratio; SD, standard deviation; SEM, standard error of the mean.

Supplementary Table 3 Studies reporting data on CVD or cardiovascular risk factors.

**Study and country**	**Population**	**AD severity/ staging measure**	**Results**
**Studies reporting data on multiple conditions/risk factors[a]**			
Eldholm et al. (2018) (13)<br>Norway	*N* = 282 with CDR 0.5, 1.0, or 2.0 (very mild, mild, or moderate AD dementia)	CDR and CDR-SB	Adjusted regression analyses showed no significant associations between any individual vascular risk factor (including hypertension, hypercholesterolemia, diabetes and Framingham Stroke Risk Profile score [assessed in men and women separately, and not adjusted for age and sex]) or history of a vascular disease (atrial fibrillation, heart disease, peripheral vascular disease or stroke or TIA), and progression of AD, including in subgroups of patients with untreated hypertension, hypercholesterolemia, or diabetes
Irimata et al. (2018) (14)<br>USA	*N* = 1,899 with AD dementia	CDR-SB	Of the cardiovascular risk factors assessed (BMI, years smoking, atrial fibrillation, diabetes, hypertension, and hypercholesterolemia), recent/active hypertension ($p = 0.0001$) and recent/active hypercholesterolemia ($p = 0.0294$) were associated with a decrease in CDR-SB score per month over an average of 52 months' follow-up; i.e., an increase in cognition
Lee et al. (2020) (15)<br>Taiwan	*N* = 330 with AD (CDR 0.5, 1.0, 2.0, or 3.0) at baseline; 226 with 3 years' follow-up	CDR-SB	There was no significant association between single VRFs (CHD, cardiac arrhythmia, cerebrovascular accident, hypertension, diabetes, obesity, smoking, and physical inactivity) at baseline and CDR-SB score decline over 3 years' follow-up<br>β (95% CI) for number of VRFs (0 VRFs as reference)<br>> 3 VRFs: 1.24 (0.53–1.95); $p = 0.001$<br>3 VRFs: 0.88 (0.21–1.56); $p = 0.01$<br>2 VRFs: 0.6 (−0.01 to 1.22); $p = 0.06$<br>1 VRF: 0.58 (−0.06 to 1.22); $p = 0.08$<br>*p* value for > 3 VRFs vs. ≤ 3 VRFs: 0.02
Mielke et al. (2007) (16)<br>USA	*N* = 135 with AD dementia with ≥ 1 follow-up; 81 with no follow-up	CDR-SB	The overall vascular index score was not associated with either CDR-SB score at baseline or with rate of decline over follow-up (mean: 3 years), but several individual vascular risk factors were associated

Study and country	Population	AD severity/ staging measure	Results
			Multivariate model controlling for age, sex, education, dementia duration, any *APOE* ε4 alleles, depression (NPI ≥ 4), plus other vascular variables<br>Atrial fibrillation<br>Coefficient (95% CI): –0.08 (–1.61 to 1.45)<br>Coefficient*time (95% CI): 1.27 (0.70–1.84); $p < 0.001$<br><br>SBP (continuous)<br>Coefficient (95% CI): 0.01 (–0.01 to 0.04)<br>Coefficient*time (95% CI): 0.01 (–0.003 to 0.01); p = NS<br><br>SBP ≥ 160 vs. < 160<br>Coefficient (95% CI): –0.06 (–1.56 to 1.45)<br>Coefficient*time (95% CI): 1.78 (1.20–2.36); $p < 0.001$<br><br>Angina<br>Coefficient (95% CI): –0.72 (–2.16 to 0.71)<br>Coefficient*time (95% CI): 0.45 (–0.02 to 0.92); $p < 0.05$<br><br>Coronary artery bypass graft<br>Coefficient (95% CI):0.61 (–1.18 to 2.40)<br>Coefficient*time (95% CI): –1.46 (–2.24 to –0.69); $p < 0.001$<br><br>Myocardial infarction<br>Coefficient (95% CI): 0.62 (–0.69 to 1.94)

**Study and country**	**Population**	**AD severity/ staging measure**	**Results**
			Coefficient*time (95% CI): 0.11 (–0.32 to 0.55); p = NS<br><br>Diabetes<br>Coefficient (95% CI): 0.57 (–0.62 to 1.77)<br>Coefficient*time (95% CI): –0.84 (–1.22 to –0.47); $p < 0.001$<br><br>Any antihypertensive medication<br>Coefficient (95% CI): 0.45 (–0.47 to 1.36)<br>Coefficient*time (95% CI): –0.61 (–1.03 to –0.19); $p < 0.01$<br><br>Stroke<br>Coefficient (95% CI): 0.75 (–0.98 to 2.47)<br>Coefficient*time (95% CI): –0.04 (–0.59 to 0.51); p = NS
Tay et al. (2019) (10)<br>Singapore	*N* = 96 (14 with MCI; 74 with AD dementia)<br>*n* = 88 with follow-up	CDR-SB	Comorbidities at baseline (all non-significant for progressors vs. non-progressors), *n* (%)<br>Hypertension<br>Progressors[b]: 11 (73.3)<br>Non-progressors: 47 (64.4)<br><br>Diabetes<br>Progressors[b]: 3 (20)<br>Non-progressors: 24 (32.9)<br><br>Hyperlipidemia

Study and country	Population	AD severity/ staging measure	Results
			Progressors[b]: 7 (46.7)<br>Non-progressors: 47 (64.4)<br><br>Ischemic heart disease<br>Progressors[b]: 1 (6.7)<br>Non-progressors: 14 (19.2)<br><br>Atrial fibrillation<br>Progressors[b]: 0<br>Non-progressors: 2 (2.7)<br><br>Stroke/TIA<br>Progressors[b]: 0<br>Non-progressors: 3 (4.1)
Yang et al. (2021) (17)<br>USA	*n* = 203 with AD<br>*n* = 158 with AD and arteriolosclerosis	CDR	Number of vascular risk factors (hypertension, diabetes, hyperlipidemia, or any smoking history), median (IQR)<br>AD only<br>CDR 0.5 (*n* = 128): 2 (1–2.75)<br>CDR 1.0 (*n* = 156): 2 (1–3)<br><br>AD with arteriolosclerosis<br>CDR 0.5 (*n* = 120): 2 (1–3)<br>CDR 1.0 (*n* = 107): 2 (1–3)

Study and country	Population	AD severity/ staging measure	Results
Yeo et al. (2013) (18)<br>Singapore	*N* = 101 with mild/moderate AD dementia	CDR-SB	The third of the cohort with the fastest CDR-SB score pre-progression had a non-significantly higher prevalence of comorbidities than the third with the slowest pre-progression<br>Hypertension: 72.7% vs. 50.0%<br>Hyperlipidaemia: 75.8% vs. 52.9%<br>Ischemic heart disease: 15.2% vs. 5.9%
**Studies reporting data on 1 condition/risk factor**			
Bleckwenn et al. (2017) (19)<br>Germany	*N* = 118 with AD or mixed dementia	CDR-SB	At baseline, patients with CHD had significantly better CDR-SB scores than those with no CHD (no data provided)<br>CHD at baseline was associated with a faster estimated annual CDR-SB decline<br>No CHD: 1.2 points<br>CHD: 2.2 points (83% faster decline)
Chou et al. (2018) (20)<br>Taiwan	*N* = 278 with AD (90 with CDR 0.5; 188 with CDR 1.0)	CDR	Proportion of patients with deterioration in CDR over 3 years' follow-up, %<br>Patients with hypertension: 44.0<br>Patients without hypertension: 34.8<br>$p = 0.13$
de Oliveira et al. (2018) (21)<br>Brazil	*N* = 191 with AD dementia	CDR-SB	β-value for the association between CDR-SB score change and variation in the 10-year absolute coronary heart disease risk over 1 year<br>Women: 0.010 (95% CI, –0.062 to 0.082); $p = 0.779$<br>Men: –0.064 (95% CI, –0.170 to 0.042); $p = 0.233$
Moon et al. (2019) (22)<br>South Korea	*N* = 69 with AD dementia	CDR-SB	Diabetes at baseline was identified as a potential covariate influencing CDR-SB score progression over 3 years' follow-up

Study and country	Population	AD severity/ staging measure	Results
Pavlik et al. (2019) (23)<br>USA	*N* = 909 with AD dementia	CDR-SB	No history of hyperlipidemia was associated with worse CDR-SB scores relative to a history of hyperlipidemia, coefficient (SE): 0.608 (0.245); $p = 0.013$

[a]Data from Chew et al. (2019) (24) found no link between CDR-SB score progression and cardiovascular risk factors. Data are not reported because the exact cardiovascular risk factors examined are not specified in the publication.

[b]Disease progression defined as an increase ≥ 2 points from baseline in CDR-SB score.

AD, Alzheimer's disease; BMI, body mass index; CDR, Clinical Dementia Rating; CDR-SB, Clinical Dementia Rating–Sum of Boxes; CHD, coronary heart disease; CI, confidence interval; CVD, cardiovascular disease; ECG, electrocardiogram; EGFR, estimated glomerular filtration rate; IQR, interquartile range; MCI, mild cognitive impairment; NPI, Neuropsychiatric Inventory; NS, not significant; SBP, systolic blood pressure; SE, standard error; TIA, transient ischemic attack; VRF, vascular risk factor.

Supplementary Table 4 Studies reporting economic data.

Study and country	Population	AD severity/ staging measure	Results
Ruokostenpohja et al. (2018) (25)<br><br>Finland	*N* = 236 home-dwelling individuals with very mild or mild AD (CDR 0.5–1.0) and their family care partners	CDR-SB	Association between score and receipt of care partners' allowance in multivariable model, OR (95% CI)<br><br>CDR-SB: 1.40 (1.30–1.50); $p = 0.000$<br><br>ADCS-ADL: 0.93 (0.92–0.94); $p = 0.000$<br><br>NPI: Model 1: 1.01 (0.99–1.03); $p = 0.437$; Model 2: 1.01 (0.99–1.03); $p = 0.434$
Jetsonen et al. (2021) (26)<br><br>Finland	*N* = 231 home-dwelling individuals with early or mild AD dementia (129 with CDR-SB 0.5–4.0, 88 with CDR-SB 4.5–6.5, and 14 with CDR-SB 7.0–9.0)	CDR-SB	Over 5 years' follow-up, a 1-unit increase in CDR-SB score was associated with increases of 15% in total care costs, 11% in formal care costs, and 18% in informal care costs<br><br>Total cost of care (annual),[a] rate ratio Exp(B) (95% CI)<br><br>CDR-SB ≤ 4: 1<br><br>CDR-SB 4.5–6.5: 1.77 (1.45–2.15)<br><br>CDR-SB 7–9: 2.76 (2.25–3.37)<br><br>CDR-SB 9.5–15.5: 3.42 (2.77–4.22)<br><br>CDR-SB ≥ 16: 4.41 (3.42–5.70)<br><br>All $p < 0.001$ vs. CDR-SB ≤ 4<br><br>Total cost of care (annual),[a] € (95% CI)<br><br>CDR-SB ≤ 4: 16,448 (13,722–19,716)<br><br>CDR-SB 4.5–6.5: 29,053 (25,091–33,642)<br><br>CDR-SB 7–9: 45,314 (38,806–52,913)<br><br>CDR-SB 9.5–15.5: 56,252 (49,451–63,990)

Study and country	Population	AD severity/ staging measure	Results
			CDR-SB ≥ 16: 72,600 (59,717–88,262)<br><br>Cost of formal care (annual),[a] rate ratio Exp(B) (95% CI)<br>CDR-SB ≤ 4: 1<br>CDR-SB 4.5–6.5: 1.57 (1.17–2.10)<br>CDR-SB 7–9: 2.34 (1.67–3.27)<br>CDR-SB 9.5–15.5: 2.42 (1.69–3.47)<br>CDR-SB ≥ 16: 3.16 (1.86–5.39)<br>All $p < 0.001$ vs. CDR-SB ≤ 4, except CDR-SB 4.5–6.5, $p = 0.003$<br><br>Costs of formal care (annual),[a] € (95% CI)<br>CDR-SB ≤ 4: 8,498 (6,480–11,143)<br>CDR-SB 4.5–6.5: 13,300 (10,145–17,437)<br>CDR-SB 7–9: 19,870 (14,103–27,994)<br>CDR-SB 9.5–15.5: 20,559 (15,461–27,338)<br>CDR-SB ≥ 16: 26,887 (16,593–43,566)<br><br>Cost of informal care (annual),[b] rate ratio Exp(B) (95% CI)<br>CDR-SB ≤ 4: 1<br>CDR-SB 4.5–6.5: 1.95 (1.48–2.56)<br>CDR-SB 7–9: 3.15 (2.41–4.13)<br>CDR-SB 9.5–15.5: 4.36 (3.34–5.68)<br>CDR-SB ≥ 16: 5.62 (4.22–7.50)

**Study and country**	**Population**	**AD severity/ staging measure**	**Results**
			All $p < 0.001$ vs. CDR-SB ≤ 4<br><br>Costs of informal care (annual),[b] € (95% CI)<br>CDR-SB ≤ 4: 8,032 (6,312–10,221)<br>CDR-SB 4.5–6.5: 15,654 (13,355–18,350)<br>CDR-SB 7–9: 25,330 (21,954–29,225)<br>CDR-SB 9.5–15.5: 34,979 (30,903–39,593)<br>CDR-SB ≥ 16: 45,169 (38,276–53,303)
Ton et al. (2017) (27)<br>USA	*n* = 121 with amnestic MCI<br>*n* = 174 with AD dementia	CDR-SB	There was a linear trend between healthcare utilization and increasingly poorer cognitive states (amnestic MCI, CDR-SB 0.5–4.0; mild dementia, CDR-SB 4.5–9.0;[c] moderate dementia; CDR-SB 9.5–15.5; and severe dementia, CDR-SB > 16.0)[d]<br><br>OR (95% CI) (normal cognition as reference category)<br>Use of home care services: 1.39 (0.96–2.01); $p = 0.084$<br>Use of nursing homes: 2.28 (1.64–3.17); $p < 0.001$<br>Hospitalizations: 1.42 (1.11–1.81); $p = 0.005$<br><br>There was a significant decreasing trend in use of outpatient services across increasingly poorer cognitive states ($p < 0.001$)<br><br>There was a non-significant linear association with doctor visits ($p = 0.064$) and drug utilization ($p = 0.130$) across increasingly poorer cognitive states in demographically adjusted models

Study and country	Population	AD severity/ staging measure	Results
			There was a trend for decreasing household income across increasingly poorer cognitive states (p < 0.015 in a model adjusted for demographics and comorbidities)
Gustavsson et al. (2011) (28)<br>Multinational	*N* = 2,744 with a diagnosis of probable AD and MMSE score 16–26; sample size for CDR-SB analysis NR	CDR-SB	Coefficient of correlation between baseline score and resource category (95% CI)<br>CDR-SB<br>Total informal care:[f] 0.415; $p < 0.001$<br>Accommodation: 0.083; $p < 0.001$<br>Hospitalizations: 0.032<br>Community services: 0.176; $p < 0.001$<br><br>ADCS-ADL score<br>Total informal care: [f] –0.475; $p < 0.001$<br>Accommodation: –0.137; $p < 0.001$<br>Hospitalizations: –0.022<br>Community services: –0.181; $p < 0.001$<br><br>NPI total score<br>Total informal care: [f] 0.249; $p < 0.001$<br>Accommodation: 0.01<br>Hospitalizations: 0.025<br>Community services: 0.074; $p < 0.001$<br><br>NPI distress score<br>Total informal care: [f] 0.234; $p < 0.001$

Study and country	Population	AD severity/ staging measure	Results
			Accommodation: –0.023<br>Hospitalizations: 0.015<br>Community services: 0.034<br><br>A one-point decrease in the ADCS-ADL score results in a 3.6% increase in total costs of care, translating into mean cost increases of:<br>Mild AD dementia: £337 (€396; $483)<br>Moderate AD dementia: £507 (€595; $727)<br>Severe AD dementia: £724 (€850; $1,039)<br>Mean increases in total costs of care per 1-point increase in NPI total score:<br>£100 (€117; $143) to £214 (€251; $307) (assumed to be across AD dementia severity categories)
Darba et al. (2015) (29)<br>Spain	*N* = 343 with AD dementia (18 with CDR 0.5, 116 with CDR 1.0, 102 with CDR 2.0, and 103 with CDR 3.0)	CDR	A 1-point increase in CDR was associated with increases of 45.8 % ($p = 0.05$) in direct medical costs, 131.2 % ($p = 0.01$) in social care costs, 1,275.7 % ($p = 0.01$) in informal care costs,[g] and 68.6 % ($p = 0.05$) in total care over 6 months, compared with the reference group (CDR 1.0)
Ikeda et al. (2021) (30)<br>Japan	*N* = 3,600,000 (estimated number of people with AD dementia in Japan in 2018)<br><br>Estimated[e] number of individuals in each CDR category:	CDR	Annual healthcare costs (excluding ADD drugs) per person with ADD (billion JPY)<br>CDR 0.5: 275,148[h]<br>CDR 1.0: 275,148<br>CDR 2.0: 419,028<br>CDR 3.0: 559,236<br><br>Annual public long-term care costs per person with ADD (billion JPY)

Study and country	Population	AD severity/ staging measure	Results
	CDR 0.5: 954,125<br>CDR 1.0: 1,151,725<br>CDR 2.0: 660,548<br>CDR 3.0: 841,211		CDR 0.5: 253,919<br>CDR 1.0: 1,151,725<br>CDR 2.0: 1,790,152<br>CDR 3.0: 2,733,045

[a]Unit costs for healthcare service use were obtained from the report on Health and Social Care Unit costs in Finland in 2011. For comparative reasons, all costs were transformed into 2016 monetary values using the national consumer price index.

[b]Informal care costs can be considered direct non-medical costs as a proxy of home care, or as indirect costs if the opportunity cost approach is used. The time spent on informal care was assessed by the opportunity cost approach in means of loss of leisure time (35% of the average Finnish gross wage per hour for lost leisure time was applied), because no data were available on productivity losses.

[c]Mild dementia is defined instead as CDR-SB 3.0–9.0 in figure legends in the publication; we have assumed that this is an error, because this overlaps with the CDR-SB score range for amnestic MCI (0.5–4.0).

[d]Adjusted for age, gender, race, education, marital status, and residential region.

[e]Estimated using the population aged 65 years and over reported in national statistics and epidemiological information reported in a previous study by Asada et al. (2013) (31) Prevalence of dementia and response to life dysfunction due to dementia in urban areas, Health Labour Sciences Research Grants, Dementia Countermeasures Comprehensive Research Project 2011-2012 Research Report.

[f]Value of lost productivity for care partners younger than 65 years, and value of lost leisure time for those 65 years or older, not including time spent on supervision.

[g]Contributed by time spent on ADLs, IADLs and supervision.

[h]Data not available for CDR 0.5; assumed by the study authors to be the same as CDR 1.0.

AD, Alzheimer’s disease; ADCS-ADL, Alzheimer's Disease Cooperative Study–Activities of Daily Living scale; ADD, Alzheimer’s disease dementia; ADL, activities of daily living; CDR, Clinical Dementia Rating, CDR-SB, Clinical Dementia Rating–Sum of Boxes; CI, confidence interval; IADL, instrumental activities of daily living; JPY, Japanese Yen; MCI, mild cognitive impairment; MMSE, Mini Mental State Examination; NPI, Neuropsychiatric Inventory; NR, not reported.

3 References

1. Barca ML, Persson K, Eldholm R, Benth JS, Kersten H, Knapskog AB, et al. Trajectories of depressive symptoms and their relationship to the progression of dementia. *J Affect Disord.* (2017) 222:146–52. doi: 10.1016/j.jad.2017.07.008
2. Caroline C, Chan PC. Baseline behavioural symptoms impact on clinical disease progression in Alzheimer's dementia. *Ann Acad Med Singapore.* (2015) 44:S340.
3. Hallikainen I, Hongisto K, Valimaki T, Hanninen T, Martikainen J, Koivisto AM. The progression of neuropsychiatric symptoms in Alzheimer's disease during a five-year follow-up: Kuopio ALSOVA study. *J Alzheimers Dis.* (2018) 61(4):1367–76. doi: 10.3233/JAD-170697
4. Wadsworth LP, Rentz D, Lorius N, Johnson K, Sperling R, Locascio J, et al. Neuropsychiatric symptoms are associated with current and future global functional impairment in mild cognitive impairment. *Alzheimers Dement.* (2011) 7:S168–9. doi: 10.1016/j.jalz.2011.05.456
5. Breitve MH, Hynninen MJ, Bronnick K, Chwiszczuk LJ, Auestad BH, Aarsland D, et al. A longitudinal study of anxiety and cognitive decline in dementia with Lewy bodies and Alzheimer's disease. *Alzheimers Res Ther.* (2016) 8(1):3. doi: 10.1186/s13195-016-0171-4
6. Hallikainen I, Hanninen T, Fraunberg M, Hongisto K, Valimaki T, Hiltunen A, et al. Progression of Alzheimer's disease during a three-year follow-up using the CERAD-NB total score: Kuopio ALSOVA study. *Int Psychogeriatr.* (2013) 25(8):1335–44. doi: 10.1017/S1041610213000653
7. Naurhashemi F, Ousset PJ, Gillette-Guyonnet S, Cantent C, Andrieu S, Vellas B. A 2-year follow-up of 233 very mild (CDR 0.5) Alzheimer's disease patients (REAL.FR cohort). *Int J Geriatr Psychiatry.* (2008) 23(5):460–5. doi: 10.1002/gps.1904
8. Jenner C, Reali G, Puopolo M, Silveri MC. Can cognitive and behavioural disorders differentiate frontal variant-frontotemporal dementia from Alzheimer's disease at early stages? *Behav Neurol.* (2006) 17:89–95. doi: 10.1155/2006/812760
9. Tschanz JT, Corcoran CD, Schwartz S, Treiber K, Green RC, Norton MC, et al. Progression of cognitive, functional, and neuropsychiatric symptom domains in a population cohort with Alzheimer dementia: the Cache County Dementia Progression study. *Am J Geriatr Psychiatry.* (2011) 19:532–42. doi: 10.1097/JGP.0b013e3181faec23
10. Tay L, Leung B, Yeo A, Chan M, Lim WS. Elevations in serum Dickkopf-1 and disease progression in community-dwelling older adults with mild cognitive impairment and mild-to-moderate Alzheimer's disease. *Front Aging Neurosci.* (2019) 11:278. doi: 10.3389/fnagi.2019.00278
11. Charernboon T, Phanasathit M. Prevalence of neuropsychiatric symptoms in Alzheimer's disease: a cross-sectional descriptive study in Thailand. *J Med Assoc Thai.* (2014) 97(5):560–5.
12. Bandyopadhyay TK, Biswas A, Roy A, Guin DS, Gangopadhyay G, Sarkhel S, et al. Neuropsychiatric profiles in patients with Alzheimer's disease and vascular dementia. *Ann Indian Acad Neurol.* (2014) 17(3):325–30. doi: 10.4103/0972-2327.138520
13. Eldholm RS, Barca ML, Persson K, Knapskog AB, Kersten H, Engedal K, et al. Progression of Alzheimer's disease: a longitudinal study in Norwegian memory clinics. *J Alzheimers Dis.* (2018) 61(3):1221–32. doi: 10.3233/JAD-170436
14. Irimata KE, Dugger BN, Wilson JR. Impact of the presence of select cardiovascular risk factors on cognitive changes among dementia subtypes. *Curr Alzheimer Res.* (2018) 15(11):1032–44. doi: 10.2174/1567205015666180702105119

15. Lee WJ, Liao YC, Wang YF, Lin YS, Wang SJ, Fuh JL. Summative effects of vascular risk factors on the progression of Alzheimer disease. *J Am Geriatr Soc.* (2020) 68(1):129–36. doi: 10.1111/jgs.16181
16. Mielke MM, Rosenberg PB, Tschanz J, Cook L, Corcoran C, Hayden KM, et al. Vascular factors predict rate of progression in Alzheimer disease. *Neurology.* (2007) 69(19):1850–8. doi: 10.1212/01.wnl.0000279520.59792.fe
17. Yang D, Masurkar AV. Clinical profiles of arteriolosclerosis and Alzheimer disease at mild cognitive impairment and mild dementia in a national neuropathology cohort. *Alzheimer Dis Assoc Disord.* (2021) 35(1):14–22. doi: 10.1097/WAD.0000000000000411
18. Yeo A, Chong MS, Tay L, Yap J, Chan M. Assessing clinical progression in Alzheimer's disease (AD) subjects: an alternative pre-progression rate in a Singapore memory clinic population. *Ann Acad Med Singapore.* (2013) 42:S268.
19. Bleckwenn M, Kleineidam L, Wagner M, Jessen F, Weyerer S, Werle J, et al. Impact of coronary heart disease on cognitive decline in Alzheimer's disease: a prospective longitudinal cohort study in primary care. *Br J Gen Pract.* (2017) 67(655):e111–e7. doi: 10.3399/bjgp16X688813
20. Chou PS, Kao YH, Wu MN, Chou MC, Chen CH, Lin RT, et al. Effect of the interaction between hypertension and cerebral white matter changes on the progression of Alzheimer disease. *Curr Alzheimer Res.* (2018) 15(14):1354–60. doi: 10.2174/1567205015666181002141013
21. de Oliveira FF, Pereira FV, Pivi GAK, Smith MC, Bertolucci PHF. Effects of APOE haplotypes and measures of cardiovascular risk over gender-dependent cognitive and functional changes in one year in Alzheimer's disease. *Int J Neurosci.* (2018) 128(5):472–6. doi: 10.1080/00207454.2017.1396986
22. Moon Y, Moon WJ, Kim JO, Kwon KJ, Joung J, Han SH. Predictors of poor clinical outcome and role of muscle profile in Alzheimer's disease: a 3-year longitudinal study. *Alzheimers Dement.* (2019) 15(7 Suppl):P700–P1. doi: 10.1016/j.jalz.2019.06.2705
23. Pavlik VN, Chan W, Darby E. Cohort effects in progression rate on cognitive and functional measures in an Alzheimer's disease clinical cohort. *J Alzheimers Dis.* (2019) 71(2):659–69. doi: 10.3233/JAD-190661
24. Chew J, Abengana J, Ali N, Chan M, Tay L, Lim WS. Self-reported sleep duration as a predictor of cognitive decline in mild cognitive impairment (MCI) and mild Alzheimer's dementia (AD). *Alzheimers Dement.* (2019) 15(7 Suppl):P1185. doi: 10.1016/j.jalz.2019.06.3589
25. Ruokostenpohja N, Valimaki T, Martikainen J, Hallikainen M, Vehvilainen-Julkunen K, Koivisto A. Entitlement of carer's allowance to support home care of persons with Alzheimer's disease: evaluation of current decision criteria. *Eur Geriatr Med.* (2018) 9(4):477–83. doi: 10.1007/s41999-018-0060-4
26. Jetsonen V, Kuvaja-Kollner V, Valimaki T, Selander T, Martikainen J, Koivisto AM. Total cost of care increases significantly from early to mild Alzheimer's disease: 5-year ALSOVA follow-up. *Age Ageing.* (2021) 50(6):2116–22. doi: 10.1093/ageing/afab144
27. Ton TGN, DeLeire T, May SG, Hou N, Tebeka MG, Chen E, et al. The financial burden and health care utilization patterns associated with amnestic mild cognitive impairment. *Alzheimers Dement.* (2017) 13(3):217–24. doi: 10.1016/j.jalz.2016.08.009
28. Gustavsson A, Cattelin F, Jonsson L. Costs of care in a mild-to-moderate Alzheimer clinical trial sample: key resources and their determinants. *Alzheimers Dement.* (2011) 7(4):466–73. doi: 10.1016/j.jalz.2010.06.002
29. Darba J, Kaskens L, Lacey L. Relationship between global severity of patients with Alzheimer's disease and costs of care in Spain; results from the co-dependence study in Spain. *Eur J Health Econ.* (2015) 16(8):895–905. doi: 10.1007/s10198-014-0642-0

30. Ikeda S, Mimura M, Ikeda M, Wada-Isoe K, Azuma M, Inoue S, et al. Economic burden of Alzheimer's disease dementia in Japan. *J Alzheimers Dis.* (2021) 81(1):309–19. doi: 10.3233/JAD-210075

31. Asada T. Prevalence of dementia and response to life dysfunction due to dementia in urban areas, Health Labour Sciences Research Grants, Dementia Countermeasures Comprehensive Research Project 2011-2012 Research Report. (2013).
